# Moderation of financial stress and cardiovascular disease risk in black catholics: A social-ecological pathway utilizing latent class and structural equation analyses

**DOI:** 10.1371/journal.pmen.0000047

**Published:** 2025-06-13

**Authors:** Marcia Elizabeth Ifeoma Uddoh

**Affiliations:** School of Medicine, All Saints University School of Medicine, Hillsborough Street Roseau, Commonwealth of Dominica; Public Library of Science, UNITED KINGDOM OF GREAT BRITAIN AND NORTHERN IRELAND

## Abstract

**Background:**

Chronic financial stress drives cardiovascular disease (CVD), yet its impact within Black Catholic communities remains understudied. Using a profile-based, socio-ecological framework, we centered chronic financial stress and low Mental Component Summary (MCS) scores, as correlates of downstream health risk, and we modeled racial discrimination as an upstream moderator that intensifies this trajectory.

**Methods:**

Cross-sectional data from 285 racially and religiously diverse adults were analyzed. Latent class analysis (LCA) identified distinct stress profiles, and structural equation modeling (SEM) tested pathways from financial stress, perceived stress, discrimination, and low MCS to a Black Catholic profile and subsequent chronic stress risk (CHRONRSK). Model fit was evaluated with χ², RMSEA, CFI, TLI, and SRMR indices; indirect effects were estimated with bias-corrected bootstraps.

**Results:**

LCA revealed three profiles—Chronic, Life Trauma, and Daily Hassles. The SEM containing financial stress and discrimination showed excellent fit (χ²(3)= 3.98, *p* = .26; RMSEA = .034; CFI = .979), whereas substituting the Perceived Stress Scale (PSS) produced poor fit (RMSEA = .131; CFI = .747). Financial stress (*β* = .124, *p* = .045) and discrimination (*β* = .227, *p* < .001) significantly predicted the Black Catholic profile, which strongly predicted chronic stress (*β* = .761, *p* < .001). Low Mental Component Summary scores neither predicted the profile (*β* = –.009, *p* = .75) nor mediated the pathway. PSS was uninformative, reinforcing that **these downstream health risks** stem from upstream structural forces, not individual psychology.

**Conclusions:**

Ninety percent (90.6%) of low-income Black Catholics fell below the national averages for Mental Component Summary scores. Chronic financial stress explained nearly half of this mental-health disparity (η² = 0.489)—evidence that upstream burdens, not individual pathology, drive downstream outcomes. For Black Catholics, chronic financial stress and racial discrimination—rather than generalized stress (PSS) or isolated mental-health scores—emerge as the most salient yet under-recognized moderators of **chronic financial-stress risk**. These findings call into question the adequacy of conventional stress models and underscore the need for upstream, equity-driven interventions. Public health strategies must advance upstream structural interventions to counter systemic forces sustaining financial stress and CVD risk.

## Introduction

Financial stress is a significant and underaddressed contributor to cardiovascular disease (CVD), stroke, and mortality [1], exerting pronounced effects in Black communities [2,3]. Among the 46.8 million Black individuals in the United States [4], many experience disproportionately high rates of cardiovascular risk factors [2,5]. Notably, Moran et al. [6] suggest that “financial stress may be an unrecognized risk factor for coronary heart disease for African Americans,” underscoring the need for additional investigations that integrate stress management and other coronary risk factors into patient interventions.

However, despite strong evidence, financial stress remains underexamined in cardiovascular disease research—particularly in studies involving Black populations. Although the Jackson Heart Study identifies financial strain as a key contributor to coronary disease within Black communities [6], many investigations examining links between financial stress and stroke, hypertension, or hyperlipidemia [7] have not accounted for subgroup-specific moderators. Similarly, models like Crosswell’s [8] framework often overlook racial context, hindering precise risk identification.

The present study addresses these gaps by placing financial stress at the center of a multifaceted approach, recognizing how structural and social determinants intersect with race to influence chronic financial-stress risk  among Black subpopulations.

Major health organizations, including the World Health Organization, have long recognized stress as a key determinant of health—yet financial stress remains systematically under-addressed in cardiovascular prevention efforts, especially among Black populations [9]. Substantial evidence indicates that many high-risk individuals—disproportionately Black—are under-identified in mainstream cardiovascular screening [10]. Each year, over one million Americans experience an initial myocardial infarction that might have been prevented through cost-effective measures [11].

The American College of Cardiology and the American Heart Association recommend stress management as part of optimal medical therapy for patients with stable coronary disease [12]. Similarly, the European Society of Cardiology designates stress management as a Class IA recommendation—its strongest endorsement—for at-risk populations. Findings from the INTERSTROKE study, a large international case-control analysis involving 13,462 cases, further underscore the importance of addressing financial stress, demonstrating that experiencing such stress in the past year significantly increases the risk for all stroke subtypes [13].

Taken together, these guidelines reinforce the urgent need to acknowledge financial stress as a pivotal yet frequently underestimated contributor to adverse cardiovascular outcomes [14]. By incorporating subgroup-specific variables—particularly those shaped by systemic inequities—healthcare practitioners and researchers can strengthen risk detection and develop tailored interventions, thereby bolstering both prevention strategies and long-term cardiovascular outcomes.

### Moderation, stress pathways, and theoretical foundations

Crosswell’s seminal work, Best Practices for Stress Measurement, introduced a framework linking stress to cardiovascular health outcomes [8]. Building on this foundation, the present study emphasizes moderation—a key concept in health psychology that clarifies how psychosocial stress translates into physiological risk [15]. Recent research underscores the interplay of biological, psychological, behavioral, and social factors. This body of work highlights the importance of moderators such as personality traits [16], social support [17], and illness-behavior dynamics [18], among others. By revealing complex interdependencies among variables, moderation analysis deepens our understanding of why standard “one-size-fits-all” measurements often fail to capture targeted risk profiles in diverse populations [15].

This study examines how financial stress—moderated by systemic factors such as discrimination and patient satisfaction—contributes to chronic financial-stress risk among Black Catholics, a historically overlooked population. A declassified Navy training document from Arlington, Virginia underscores the need for a “conceptual understanding of how individual difference variables influence the stress-performance relationship … to make better predictions” [19]. This directive aligns with similar concerns raised by de las Fuentes [20], a former American Psychological Association president, who advocates for “structural competence” in recognizing systemic barriers—including poverty, discrimination, and unequal resource access—that shape mental and physical health outcomes. Earl et al. [21] likewise observe that within-group diversity remains inadequately considered in many studies of Black Americans, noting a tendency to treat this population as homogeneous.

Building on Crosswell’s [8] stress-to-cardiovascular-disease model, this study introduces moderation as a key strategy for identifying high-risk Black subpopulations. Grounded in a social-ecological perspective [22,23], it examines how stress operates across multiple levels—from individual to policy. Indeed, Crosswell’s framework posits that heightened stress—particularly when coupled with low mental component scores—correlates with elevated cardiac risk [8]. However, initial empirical applications in this study revealed discrepancies: the proposed stress timeline did not align with expected **chronic financial-stress risk** outcomes among Black Catholics, suggesting that more rigorous validation is needed to ensure subgroup-specific timelines align with real-world cardiovascular outcomes.

Discrimination is a critical moderator in stress-related cardiovascular outcomes. Brewer describes racial discrimination as an “environmental stressor” that operates at individual and institutional levels, triggering chronic neuroendocrine responses and heightening systolic blood pressure in Black—but not White—patients with hypertension [24]. Similarly, Wyatt [3] embeds discrimination within a socio-ecological model that highlights institutional and relational factors influencing cardiovascular health [3]. This pathway is further supported by evidence linking perceived racism to elevated CVD risk in historically marginalized groups [24].

Collectively, these findings underscore the need to incorporate culturally relevant moderators—such as discrimination, patient satisfaction—into frameworks assessing financial stress and cardiovascular disease. By integrating moderation techniques and acknowledging within-group diversity, future research will be better positioned to detect, monitor, and address the complex pathways that lead to adverse cardiac outcomes in underrepresented subpopulations, particularly Black Catholics.

### Theoretical foundations: Stress theory and the social-ecological model I

#### Allostatic load and financial stress.

The Black Cardiovascular Ecological (BaCE) pathway is anchored in the concept of allostatic load, which represents the cumulative wear and tear on the body resulting from chronic stress. Building on Pearlin’s seminal work [25], Kahn and Pearlin [26] demonstrated through the Alameda study that persistent financial strain compounds additional stressors, heightening allostatic load and undermining the body’s resilience [27]. This dynamic underscores the need to address the fundamental causes of financial stress [26], particularly in populations exposed to discrimination or low patient satisfaction, [28–30]. Pearlin [25] further emphasizes mediation in stress theory, positing that mediating variables—such as discrimination or patient satisfaction—directly influence health trajectories.

#### Need for precise stress measures.

Despite the broad application of stress theories, accurately measuring stress within specific subpopulations remains challenging [25]. This concern is especially salient for Black individuals disproportionately affected by cardiovascular disease, where precise measures of financial stress can enable earlier screening for high-risk CVD patients [28]. Brief, validated self-report tools have proven effective for detecting stress- and distress-related indicators [31]; however, these tools must evolve to better reflect the lived experiences and structural contexts that uniquely shape stress in minoritized groups.

#### Social-ecological model.

In tandem with stress theory, the social-ecological model provides an organizational framework spanning individual, interpersonal, community, and policy levels. Pearlin [25] emphasizes that mental health is influenced by broader societal structures, aligning with research initiatives [32] that underscore community and policy interventions. For instance, Balcázar et al. [33] eight-year project—funded by the National Institute for Minority Health and Health Disparities—developed the My Heart, My Community curriculum to address cardiovascular health through a holistic social-ecological perspective. This initiative highlighted the often-overlooked policy tier by advocating for community health workers and systemic reforms, underscoring the critical role of community health infrastructure in advancing cardiovascular equity [33].

Although multiple studies employ the social-ecological model to mitigate cardiovascular disease [23], many focus solely on individual-level interventions and overlook broader societal drivers. Pahn’s [22] multilayered research indicates that prevention behaviors improve when social-ecological factors are addressed collectively. Yet the policy level frequently remains absent [34], limiting the model’s potential to address systemic barriers. Pimple’s [35] exploration of psychological phenotypes similarly underscores the value of a social-ecological framework for cardiovascular disease prevention. By examining stress and mental health across these tiers, researchers can pinpoint critical intervention opportunities—particularly for Black subpopulations bearing disproportionate financial burdens and health disparities. This study builds on these findings by applying a fully tiered social-ecological framework lens to stress measurement and cardiovascular risk identification for chronic financial stress profiles among Black subpopulations.

### Theoretical foundations: stress theory and the social-ecological model II

#### Pathway moderation model approach.

Our model integrates multiple empirically supported moderators that alter the relationship between low mental component scores (MCS) and high stress—two key cardiovascular risk factors [8,36,37]. Moderators influence the strength or direction of these risk relationships, enabling more precise targeting in interventions [15]. By pinpointing these variables, practitioners can better identify individuals most in need of interventions—departing from conventional stress measurement approaches that focus predominantly on individual-level factors [34]. Instead, our innovative BaCE pathway offers a broader framework that extends beyond individual-level interventions to explore cardiovascular-risk factors at each level of the social-ecological model. This multi-tiered perspective clarifies how systemic, environmental, and social factors shape cardiovascular disease progression, laying the groundwork for targeted, equity-driven interventions.

#### Broadening traditional screening.

Traditional cardiac risk models frequently address stress and mental health but often fail to incorporate moderators that could tailor risk assessments for specific subgroups. Although some research examines factors such as neighborhood poverty [38], discrimination [39], and life stressors [40], further refinement is required to capture the contextual complexities of Black communities. MacKinnon [15] observes that the adaptability of moderators is particularly important for illuminating how diverse subpopulations experience and respond to stress, offering a more precise method of identifying cardiovascular risk pathways.

#### Structural equation modeling.

We used structural equation modeling (SEM), a multivariate technique for analyzing complex variable relationships [41], to evaluate these effects. SEM provided a comprehensive framework for testing how potential moderators—religious affiliation, patient satisfaction, and discrimination—shape chronic financial-stress risk within the social-ecological model’s five tiers. Here, MCS and financial stress function as the central variables, while integrating these moderators enhances the precision of stress-related risk detection among Black Catholics.

### Black Catholic populations and unaddressed stressors I

The model requires further refinement at the individual level of the social-ecological framework. Here, personal identity can be explicitly analyzed—an especially important consideration for culturally specific research. Mental health in Black subpopulations, particularly among Black Catholics, reflects an intersectional identity that may elevate cardiovascular risk [42]. Originating from Black feminist studies, intersectionality posits that experiences cannot be fully understood through race alone [43]. Over time, this framework has expanded to illustrate how race and other intersecting factors intensify mental health challenges [32]. By adopting this concept, the present study isolates unique stressors impacting Black Catholics and offers greater precision in identifying risk factors for this subpopulation [44]. Critically, the interplay between cardiovascular disease and these overlapping social identities underscores how cumulative effects at intersectional points can amplify health risks.

Research shows that individuals may hold multiple intersecting identities, each contributing to unique vulnerabilities [45]. These overlapping identities can amplify cardiovascular risk, as additional moderators often arise at these junctures [42]. However, many cardiovascular health models continue to treat Black populations as monolithic, overlooking how intersectionality can activate distinct risk pathways. Empirical evidence consistently indicates that single-factor analyses fail to capture significant mental health disparities within Black subgroups experiencing financial stress [21]. This oversight limits our ability to detect subtle disparities and design truly effective interventions.

#### Why focus on Black Catholics?

The first African American community was also a Catholic community [46].

Historical records confirm that Black Catholics have been an integral part of the Church’s history in America since the 16th century. As Davis Cyprian [46]explains:

“The first Roman Catholics to walk on American shores were Spanish speaking, and they were White and Black. The Spanish arrived in Florida in 1565. Many of the Blacks were slaves; others were free. From the sacramental registers one is able to identify and describe this first African American community which was also a Catholic community.

Sacramental registers have been kept in Catholic parishes since at least the 15th century. As a result, we have a display of the Black Catholic community more detailed in many instances than the official census [46].”

#### Historical foundation.

The selection of Black Catholics is well-grounded in their rich history and cultural significance within the U.S. Catholic tradition, where they constitute a distinct and enduring community [46]. Today, Black Catholics number over three million in the United States, yet this community is often misunderstood or treated as peripheral within Catholic scholarship [47]. This historical foundation challenges the misconception that Black Catholicism is a recent or marginal phenomenon; instead, it is integral to understanding the American Catholic experience. As Herman et al. [48]observe, “African Americans as a population show the sustained ability to survive an evolving array of social, economic and environmental adversities that date back to more than a century before the founding of the United States.”

### Black Catholic populations and unaddressed stressors II

Despite their longstanding presence, contemporary discussions on Black Catholics often neglect the community’s distinctive challenges and contributions, particularly concerning financial stress and health inequities. Research indicates that Black individuals with a profound sense of history and communal identity can exhibit resilience to financial stress, influenced by an acute awareness of collective struggles [28]. This resilience is especially pronounced among Black Catholics, whose early communities helped establish some of America’s earliest Catholic settlements [46]. Although marginalized, Black Catholics have wielded significant influence in shaping American history. Notably, they played a pivotal role in founding Fort Mose in 1738—the first free Black town in the United States [49].

#### Contemporary relevance.

Today, Black Catholics occupy a distinctive position within the broader Black American experience, negotiating intersecting challenges related to race and religion [47]. By foregrounding this subpopulation, the present study aims to advance financial stress research through a more contextualized understanding of the historical, social, and spiritual factors that uniquely shape Black Catholics’ lived experiences.

#### Why traditional religious buffers may fail Black Catholics.

While religious affiliation is often associated with mental and financial stress buffering [50,51], this protective effect may not fully extend to Black Catholics. One key reason is that Black Catholics are frequently marginalized within their own religious communities, leaving their struggles deprioritized [52]. Pew Research [53] indicates that only 25% of Black Catholics attend predominantly Black churches—meaning the vast majority worship in settings where their specific concerns may not be reflected in church leadership or community support. This gap is further evidenced by travel patterns: “Black Catholics also travel farther to get to their services – 41% of Black Catholic attenders report traveling more than 15 minutes to get to their parish, compared with smaller shares among White (26%) and Hispanic (30%) Catholics [53].”

#### Cultural disconnect and institutional racism.

This physical distance underscores a broader challenge: Black Catholics often lack not only geographic proximity but also consistent institutional support from community-based church structures that have historically provided resources [54]. Many Black Catholics have described the Catholic Church as “simultaneously both a loving home and a significant source of suffering because of racism” [54].

#### Cone’s theological critique.

Cone—widely recognized as a foundational figure in Black liberation theology—critiques the Catholic Church for failing to engage racism as a theological issue [55]. In the absence of institutional advocacy or theological engagement with race-based stressors, Black Catholics may face unique barriers in accessing the social and financial support systems that benefit other Catholic populations.

### Black Catholic populations and unaddressed stressors III

#### Institutional exclusion in research.

Although Black Catholics have long advocated for institutional recognition within the Church—exemplified by the 1968 formation of the Black Catholic Clergy Caucus [55]—they have historically been excluded from major health studies [56]. In 1968, the National Institutes of Mental Health undertook a notable investigation acknowledging sociologist Joe Feagin’s concerns about the neglect of Black Roman Catholics in health research [56]. Despite this early recognition, limited progress has been made in addressing health outcomes among Black Catholics, a challenge compounded by the U.S. Census’s discontinuation of religious affiliation data collection after 1890—ironically coinciding with the rise of systematic health data [57]. The absence of such data continues to obscure the interplay of race, religion, and socioeconomic status in shaping health outcomes.

#### Study’s contribution.

This study endeavors to bridge decades of oversight by aligning historical and contemporary data on Black Catholics to elucidate their mental health disparities, especially as they relate to religious affiliation, patient satisfaction, and discrimination within the context of financial stress. Pew Research indicates that religious identity and experiences of discrimination are particularly salient for Black Catholics [53]. These findings underscore the importance of addressing such moderators in mental health research. By illuminating these dynamics, our analysis offers an innovative perspective on how financial stress influences mental health outcomes in a historically understudied community, leveraging their extensive historical presence to inform and advance equitable health interventions.

#### Black Catholics and intersectionality.

Stress theory posits that stress measurement must be tailored to individual experiences [25]. Yet many studies evaluate Black populations as a monolithic group, overlooking the varied stress profiles present in distinct subgroups [21]. Census data further reveal that the fastest-growing racial or ethnic group in the United States is individuals identifying with two or more races [58]. Meanwhile, Pew Research notes a decrease in the proportion of people who self-identify solely as “Black” [4]. These shifts underscore the need to evaluate cardiovascular risk using subgroup-specific models that reflect evolving identities and lived experiences.

#### Subgroup-specific investigation.

Focusing on moderators, this study centers on low-income Black Catholics, whose mental health outcomes reflect the intersection of financial stress and socio-religious factors [21]. By delineating Black Catholics as a distinct analytic group, we can identify subgroup-specific moderators that significantly influence mental health, thereby paving the way for targeted and contextually relevant interventions. Employing an intersectional lens goes beyond race alone, highlighting how religious affiliation and healthcare satisfaction—both independently and collectively—shape mental health experiences within this population.

## Rationale for this study

Existing stress-to-cardiovascular-disease models frequently prove inadequate when applied to Black subpopulations, particularly Black Catholics. Most frameworks treat stress as a uniform risk, overlooking moderators such as religious affiliation, patient satisfaction, and discrimination—factors that can profoundly shape health outcomes in ways traditional, non-moderated models fail to detect. By refining Crosswell’s [8] stress-related pathway to cardiovascular disease with a moderation approach, this study aims to more accurately identify high-risk individuals in diverse Black communities, thereby improving both screening accuracy and targeted interventions [22,23].

### Hypothesis and validation

We hypothesize that financial stress exerts a primary influences **chronic financial-stress risk** among Black subgroups, but its impact is moderated by subgroup-specific variables functioning at multiple social-ecological levels (e.g., religious support, institutional structures, systemic barriers). When these moderators are overlooked, existing models risk misclassifying high-risk individuals, which increases the likelihood of delays in preventive care [31]. Indeed, our empirical application of Crosswell’s [8] framework revealed a mismatch in predicting cardiac disease among Black Catholics, suggesting that further validation is required to account for subgroup differences in stress timelines. This has critical implications for designing timely, equitable interventions.

#### Novelty and Contribution.

This study represents the first focused investigation into the mental health of Black Catholics—an understudied group with deep historical and cultural roots. By integrating moderation techniques within a social-ecological framework, our approach addresses the interplay of financial stress, systemic factors, and psychological responses, offering new insight into stress-related risk pathways and guiding more effective health strategies.

## Methods

### Context and settings

CloudResearch is a well-established academic organization that specializes in enhancing academic research studies using Amazon Mechanical Turk (MTurk). It provides innovative features such as feasibility calculators, eligibility controls, attention and engagement checks, and geolocation blocking, which improve data quality and participant selection on MTurk [59]. CloudResearch has been widely adopted by over 6,000 labs and researchers, demonstrating its significant impact on the field [59]. As an online labor market, MTurk has become increasingly popular among social scientists as a source of survey and experimental data. According to a study by Farrell and Sweeney [60], approximately 32,000 papers containing the phrase “Mechanical Turk” were published between 2015 and 2019, and many of these papers have been published in top-ranked social science journals [60].

### Sample

#### Participants.

Data were collected through an internet-based self-report survey instrument, utilizing a nationwide sample of 285 low-income respondents across the United States. The recruitment and survey administration were conducted via CloudResearch on the MTurk platform, specifically targeting adults residing in the U.S. whose income levels did not exceed the federal poverty guidelines [61]. Eligibility criteria were informed by the national poverty threshold as determined by the U.S. Census Bureau, which guides eligibility for federal programs such as Medicaid, CHIP, and Marketplace insurance plans; individuals were classified as low-income if their reported income was at or below 150% of the FPL [61]. We focused on low-income individuals to better understand the relationship between income, financial stress, and mental health outcomes. The survey was administered over a period of four days, from July 30, 2021, to August 2, 2021, and was designed to include adults aged 18 and above from various life stages, to ensure a comprehensive analysis of the impacts of financial stress across a broad spectrum of the population.

#### Power analysis.

A power analysis was conducted with GPower 3.1 to determine the adequacy of our sample size. The analysis was based on a moderate effect size of 0.5, a significance level (alpha) of 0.05, and a desired power level of 0.95. The results indicated that a minimum sample size of 111 participants would be required to detect meaningful effects. With a total sample size of 285 participants, our study exceeds this requirement, ensuring sufficient power to detect significant differences and interactions within the data.

### Ethics and approvals

The study procedures, including the consent process, were conducted online and approved by the Ethics Committee at All Saints University School of Medicine. Participants received detailed information about the study’s objectives, methods, potential risks, and benefits prior to participation. Informed consent was obtained electronically; participants had to click a button to confirm their understanding and voluntary agreement to participate. To ensure confidentiality, each participant was assigned a randomized ID number by MTurk, preventing access to personal identifying information by the research team. It is important to note that MTurk does not provide documentation on recruitment. The study was conducted in accordance with the Declaration of Helsinki, and all ethical guidelines were strictly followed. Documentation related to ethics approval, including the approval number and detailed ethical considerations, was submitted separately as required by the journal’s guidelines.

### Measures

#### Financial anxiety scale.

The Financial Anxiety Scale, developed by Archuleta [62], is a validated instrument designed to quantify an individual’s financial stress. It comprises seven items rated on a 7-point Likert scale. The scale demonstrates excellent internal consistency, with a Cronbach’s alpha of 0.94, indicating high reliability [62]. This scale was selected due to its demonstrated correlations with the 12-Item Short Form Survey (SF-12) Mental Component Summary (MCS) and Physical Component Summary (PCS), as well as key variables within our PROCESS macro model.

#### SF-12 health survey.

The SF-12 Health Survey is a concise yet effective tool for assessing health outcomes, particularly physical and mental health [63]. It serves as a compact version of the SF-36 Health Survey, retaining its reliability and validity in diverse settings. Studies, including those by Shah and Brown [63], have confirmed the SF-12’s reliability for evaluating health outcomes. The SF-12’s validity has been rigorously established, reflected by Cronbach alpha scores of 0.87 for PCS and 0.86 for MCS, underscoring its utility in health research [63]. The SF-12, which includes the Mental Component Summary score, was included to assess stress-related risk, as prior research has linked both stress levels and mental health to cardiovascular disease outcomes [8].

#### Perceived stress scale (PSS-10).

In addition, we administered the Perceived Stress Scale (PSS-10), developed by Cohen et al. [64], which is a widely utilized instrument for measuring generalized stress. It employs a 5-point Likert scale ranging from 0 (never) to 4 (very often) to capture respondents’ perceptions of stress over the past month. The scale has demonstrated strong internal consistency with a Cronbach’s alpha coefficient of 0.84, affirming its reliability in stress assessment [64]. The Perceived Stress Scale was included to assess whether generalized stress or the more specific financial stress construct served as a better indicator of chronic stress within our structural equation model for the target population.

#### Everyday racial discrimination scale.

Additionally, participants completed the Everyday Racial Discrimination Scale, validated by Williams et al. [65], which assesses the frequency of chronic racial discrimination experiences. This scale consists of nine items and uses a 5-point Likert scale for responses, with options spanning from “almost every day” to “never.” Previous studies have confirmed the reliability of this scale, evidenced by a Cronbach’s alpha coefficient of 0.84 [65]. It is designed to measure the pervasive nature of discriminatory experiences, making it a critical tool in studies exploring racial disparities.

#### Multidimensional scale of perceived social support.

The MSPSS is a multidimensional scale [66] that measures perceived social support and is widely used in psychological research with strong psychometric properties. The scale validated in numerous studies and shows a consistently high internal reliability across various studies. One study reported Cronbach alpha of 0.96 and 0.97 for the subscales [67].

The MSPSS includes a three-factor structure with measurement of perceived social support from Family, Friends, and Significant Other subscales [66]. Respondents rated social support measures on a Likert scale with seven points (strongly disagree [1] to strongly agree [7]). The Multidimensional Scale of Perceived Social Support was included to assess whether social support mitigated the negative effects of financial stress within the study population.

#### Neighborhood problems index.

The Neighborhood Problems Index assesses the respondent’s neighborhood [68]. The index has 10 questions on problems experienced in the neighborhood that respondent’s rate as 1, not a problem, 2, some problem, or 3, a serious problem. The Cronbach alpha for this study was 0.79 [68]. These findings suggest that persistent neighborhood challenges may serve as chronic stressors, potentially contributing to adverse health outcomes.

Therefore, the connection of poor health and neighborhood was an important factor to analyze as we aimed to determine the place of the neighborhood to exacerbate as an external stressor to subgroups of Blacks to determine the impact on mental health.

#### Patient satisfaction questionnaire.

The Patient Satisfaction Questionnaire (PSQ) assesses patient satisfaction in healthcare Settings. While an 80-item version (PSQ-III) exists, the 18 item (PSQ) scale was used, which correlated with their attributes [69]. This scale demonstrates acceptable internal reliability [70].

Respondents rate each statement on a 5-point scale ranging from 1 (strongly agree) to 5 (strongly disagree). Higher scores reflect greater satisfaction with medical care [70]. This assessment is the most widely tested instrument on patient satisfaction. Research has shown that “measuring and implementing changes based on patient satisfaction questionnaires can improve patient outcomes” [71]. Relative to our study, we were interested in the subgroups as the relative satisfaction a person has with their healthcare could reflect a correlation with their health outcome. As our group of Black Catholics experience lower levels of Mental Component Summary scores, we wanted to see how their satisfaction of their health was or was not correlated with this measure. This is because Black Catholics have a more focused concern for discrimination than most Black Americans [53], and we wanted to see if this variable could influence their health outcomes based on any perceptions they had. One study revealed that, in a multivariate analysis, being non-White was the strongest predictor of several subscales on the PSQ-III satisfaction measure, even after controlling for factors like age, gender, marital status, education, cancer site, illness duration, and metastatic status [71]. This finding was significant, leading the researcher to emphasize the need for health care systems to become more responsive to the needs of individuals from diverse ethnic backgrounds and varying levels of acculturation [71].

#### Religious affiliation.

Assessment of religious affiliation was ascertained with the question, “What is your current religion, if any?” Initially presented with a comprehensive list of twelve possible religious affiliations, participants were invited to identify their affiliation from a spectrum that included diverse religious groups such as Christian Protestant, Catholic, Atheist or Agnostic, and Nothing in Particular, along with a provision for specifying an “Other” affiliation not explicitly listed.

For analytical considerations, this study focused predominantly on the Christian Black demographic, engaging in interdenominational comparisons within Christianity. Responses were strategically categorized into a “Four-Group Basic” framework: “Black Christian Protestant,” “Black Catholic,” “Black Atheist or Agnostic,” and “Black Nothing in Particular.” This categorization facilitated an improved analysis of religious affiliations, enhancing our understanding of variations and commonalities across Black Christian denominations. The inclusion of “Black Atheist or Agnostic” and “Black Nothing in Particular” alongside Christian denominations was crucial for contextualizing Black Christian beliefs within the broader array of religious and non-religious identities, thus broadening the comparative scope of our study.

#### Single item question.

We included a single-item question in the study to determine the timeline for the onset of participants’ stress [8]. Crosswell [8] notes that stressor exposures can be measured through various approaches, including self-report questionnaires, interviews, and objective assessments. Given this flexibility in measurement, single-item questions focusing solely on identifying a timepoint are a recognized method that does not necessitate additional validation [8]. This approach aligns with established research practices, as demonstrated in Crosswell’s [8] article, which examined similar self-reported measures to capture timing-related information.

We followed **Huang’s** [72] **multi-step process** to validate all the single-item questions. First, **content validity** was established by researching comparable validated items. Next, **criterion validity** was assessed by comparing the single-item questions to the validated **Perceived Stress Scale (PSS)** using **Analysis of Variance (ANOVA)** in **SPSS** IBM SPSS Statistics for Windows, version 28 (IBM Corp., Armonk, NY, USA) [73].

Although test-retest reliability is often used to further assess single-item measures, we were unable to conduct this step due to the anonymous nature of our online survey, which did not allow for participant follow-up. However, we followed the multi-step validation process recommended by Huang [72], completing all steps except test-retest. To address this limitation, we relied on Matthews et al. [74], who provided large-scale empirical evidence that single-item measures can reliably and validly assess various constructs. Their findings support strong content and criterion validity, minimal usability concerns, and moderate to high test-retest reliability across a wide range of domains [74]. Given this established body of evidence, our approach aligns with best practices for utilizing single-item measures in applied research.

## Data analysis

Descriptive statistical analysis was conducted to assess the distribution of MCS, financial stress, and perception scores across the study population. This analysis included determining the frequency of participants whose MCS fell below or above the national average of 50, as indexed in the National Longitudinal Survey of Youth 1979 (NLSY79) [75]. The results of this analysis offer detailed insights into the variability and distribution of MCS scores among the participants.

### Group mean discrimination scores in Black religious affiliations

This study examined differences in perceived discrimination among Black individuals categorized by their religious backgrounds. The analysis focused exclusively on participants who identified as Black, drawn from a larger dataset.

#### Measures.

Perceived discrimination was assessed using D_Total (Discrimination Total Short), a measure of self-reported experiences of discrimination. The independent variable was religious affiliation, categorized into distinct groups within the Black community.

#### Analytical approach.

The analysis calculated mean discrimination scores across the different religious groups to evaluate potential differences in perceived discrimination. Descriptive statistics, including group means and sample sizes, were examined to compare variations in reported discrimination among Black individuals from diverse religious backgrounds.

#### Paradox assessment.

This study assessed mental and physical health outcomes using validated survey instruments. Mental health was measured using the Mental Component Summary, and physical health was assessed using the Physical Component Summary, both derived from the SF-12 Health Survey, a widely used instrument for evaluating health-related quality of life.

Financial stress was measured using the Financial Anxiety Scale, which assesses the psychological burden associated with financial stress. Discrimination was quantified using the Everyday Racial Discrimination Scale, a validated measure of chronic exposure to discriminatory experiences.

#### Analytical approach.

The analysis focused on within-group comparisons, examining the impact of financial stress and discrimination on mental and physical health outcomes among individuals from diverse backgrounds, including Black Catholics. Descriptive statistics were used to assess MCS, PCS, financial stress, and discrimination scores across different groups. The study also explored potential health paradoxes, particularly among low-income Black Catholics, to determine how intersecting identities may shape health outcomes.

### Correlation between financial stress and mental health among Black individuals

A Pearson correlation analysis was conducted to examine the relationship between financial stress and mental health outcomes among Black individuals, including those who identify as Black Catholics. The analysis was performed on a subset of Black individuals drawn from a larger dataset.

#### Measures.

The dependent variable (DV) was the Mental Component Score (MCS), a continuous measure of mental health. The independent variable (IV) was financial stress, measured using the Finance Score RAW variable, which captures levels of self-reported financial stress.

#### Analytical approach.

A Pearson correlation coefficient (r) was calculated to assess the strength and direction of the relationship between financial stress and MCS scores.

### Impact of financial stress on life by profile for Black Catholics with chi-square analysis

We performed a cross-tabulation analysis to examine the association between financial stress timelines and latent profiles among Black Catholics. The financial stress categories were based on Crosswell’s timescale [8] framework, which outlines stress impacts from chronic to acute stress. Each respondent was classified into a profile based on their primary stress timeline, with data analyzed using chi-square tests to assess differences between profiles. This approach enabled us to evaluate how the timeline mapped to each profile’s health measures and **financial-stress risk**.

#### Social-ecological cross-tabulation.

The aim of this portion of the study was to analyze the sources of financial stress across various domains—personal, relational, institutional, communal, policy-based, and legal—among different religious groups, including Catholics. This analysis sought to understand unique patterns of stress and their implications on the affected populations. Participants responded to the question, “Thinking about your FINANCES, which area causes you the most STRESS?” We organized the responses in a cross-tabulation by religious affiliation to examine the distribution of financial stressors. A chi-squared test was applied to confirm the statistical validity of the observed distributions.

### Study design and participants

A chi-square analysis was conducted to examine the relationship between financial stress levels and perceived sources of financial stress among Black Catholics. The analysis included only participants who identified as Black Catholic, drawn from a larger dataset.

#### Measures.

The independent variable (IV) was financial stress level, categorized into low financial stress (Quartiles 1 & 2) and high financial stress (Quartiles 3 & 4). The dependent variable (DV) was the perceived primary source of financial stress, classified into five categories: personal, relational, institutional, community, and policy-related stressors.

#### Analytical approach.

A Pearson Chi-Square test of independence was performed to assess whether the distribution of perceived financial stress sources differed significantly between individuals with low vs. high financial stress. Expected and observed frequencies were analyzed to determine whether individuals with higher financial stress systematically attributed stress to different sources compared to those with lower financial stress. Assumption checks were conducted to evaluate cell frequencies, and additional measures of association (such as Pearson’s *r* and Spearman’s correlation) were calculated to assess the strength and direction of the relationship.

### ANOVA: financial stress and mental health among Black Catholics

This study employed a one-way analysis of variance (ANOVA) to examine the relationship between financial stress and mental health outcomes (MCS scores) among Black Catholics. The analysis focused exclusively on Black Catholics, who were selected from a larger dataset (*N* = 285).

#### Measures.

The dependent variable (DV) was the MCS, a continuous measure of mental health. The independent variable (IV) was financial stress, categorized into distinct levels to assess its impact on MCS scores.

#### Analytical approach.

A one-way ANOVA was conducted to determine whether financial stress levels significantly influenced MCS scores. The analysis was performed using IBM SPSS Statistics. Assumption testing was conducted to verify the validity of the ANOVA, including:

Levene’s Test for homogeneity of variance, ensuring equal variance across groups.

Assessment of normality, using histograms and descriptive statistics.

In cases where significant differences were detected, effect size estimates were calculated, including eta-squared (η²), epsilon-squared, and omega-squared to quantify the proportion of variance explained by financial stress. All statistical tests were conducted at a 95% confidence level, with significance set at *p* < .05.

### ANCOVA: effects of contextual stressors and religious affiliation on mental health (MCS)

A univariate ANCOVA was conducted to explore the influence of religious affiliation and other contextual factors on MCS among Black participants. The larger dataset included 285 participants; however, this analysis focused exclusively on those identifying with one of four Black religious groups (Black Protestant, Black Catholic, Black Atheist/Agnostic, Black Nothing in Particular). MCS served as the dependent variable, while independent variables included neighborhood stress (NeSTR_av), social support stress (SoSTR_av), patient satisfaction (MdSTR_rev), and discrimination (D_Total). Religious affiliation was included as a between-subjects factor. The analysis tested whether these factors significantly impacted MCS and if religious affiliation moderated these relationships. The ANCOVA used Type III Sum of Squares with a significance level set at p < .05.

### One-way ANOVA: patient satisfactions care by MCS

A one-way ANOVA was performed to investigate the relationship between Mental Component Summary score MCS and the patient satisfaction in a sample of 285 participants. The sample was divided into two groups: participants with below-average MCS (*N =* 226) and those with above-average MCS (*N* = 59), based on national norms for mental health (NLSY79_MCS_Below_50). Patient satisfaction was measured using a reverse-coded version of the Patient Satisfaction Questionnaire, where higher scores indicated more negative perceptions of healthcare services. The analysis tested for differences in patient satisfaction between these two groups, and effect sizes were calculated using Eta-squared to assess the strength of the relationship.

### Two-way ANOVA: financial stress and religious affiliation effects on mental health

A two-way ANOVA was employed to explore the relationship between financial stress and religious affiliation on Mental Component Summary score MCS scores. The sample consisted of 259 participants from various religious groups: Protestant, Catholic, Atheist/Agnostic, and Nothing in Particular. Financial stress was categorized into three levels: low, medium, and high, and MCS was used as the dependent variable.

The analysis assessed the main effects of financial stress and religious affiliation, as well as their interaction on MCS. Post-hoc Bonferroni tests were conducted to identify significant pairwise comparisons between the financial stress groups. Additionally, comparisons were made between Black Catholics and other religious groups to examine potential differences in mental health outcomes.

### Mediation analysis: financial stress as a mediator between black catholic identity and mental health

This study employed a mediation analysis using the PROCESS macro (version 4.2) developed by Andrew F. Hayes [76] in SPSS to examine whether financial stress (M: raw_fin) mediated the relationship between Black Catholic identity (X: C_BAfr) and mental health (Y: Mental Component Score, MCS). The analysis focused exclusively on Black Catholics, who were selected from a larger dataset (*N* = 285).

#### Measures.

The independent variable (X) was Black Catholic identity (C_BAfr), treated as a categorical predictor. Financial stress (M: raw_fin) was included as a continuous mediator, capturing self-reported financial strain. The dependent variable (Y) was Mental Component Score (MCS), a measure of mental health.

#### Analytical approach.

A mediation model (PROCESS Model 4) was estimated to assess both direct and indirect effects of Black Catholic identity on mental health, with financial stress as a mediator. The analysis was conducted in SPSS, using 5,000 bootstrap resamples to generate bias-corrected confidence intervals (CIs) for indirect effects.

Regression coefficients (*b*), standard errors (*SE*), and 95% confidence intervals (CIs) were reported for both the direct effect of Black Catholic identity on mental health and the indirect effect through financial stress. Model fit and significance levels were evaluated using R² and F-statistics to determine the proportion of variance explained in the outcome variable.

### PROCESS model 1: Catholic affiliation moderating financial stress and MCS

We conducted a moderation analysis using the PROCESS macro to examine whether Catholic affiliation moderated the relationship between financial stress (X: FinSTRav) and mental health (Y: Mental Component Score, MCS). Model 1 was specified, with financial stress as the independent variable, Catholic affiliation as the moderator, and MCS as the dependent variable.

A subset of participants who identified as Catholic was analyzed from a larger dataset of *N =* 285. Bootstrapping with 5,000 resamples was applied to generate bias-corrected confidence intervals (CIs) for the interaction effects. The analysis included an interaction term (FinSTRav × Catholic) to test whether the effect of financial stress on mental health differed based on Catholic affiliation.

Regression coefficients, standard errors (SE), and significance levels were reported for both direct and interaction effects. Model fit was assessed using R² change statistics to determine the contribution of the interaction term to the variance explained in MCS.

### Mediation analysis: financial stress as a mediator between discrimination and the Black Catholic chronic profile

We conducted a mediation analysis using the PROCESS macro to examine whether financial stress (M: FinSTRav) mediated the relationship between discrimination (X: D_Total) and the Black Catholic Chronic Profile (Y: Pro1_BkC). Model 4 was specified, with discrimination total as the independent variable, financial stress as the mediator, and the Black Catholic Chronic Profile as the binary outcome variable.

A subset of Black Catholics classified as “chronic” was analyzed from a larger dataset (N = 285). Given the binary nature of the outcome, logistic regression was employed, and all effects were reported in a log-odds metric. Bootstrapping with 5,000 resamples was used to estimate indirect effects with bias-corrected confidence intervals (CIs).

Regression coefficients (*b*), standard errors (*SE*), and 95% CIs were reported for both direct and indirect effects. Model fit was assessed using -2 log-likelihood (-2LL), McFadden’s R², Cox & Snell R², and Nagelkerke R² to evaluate the strength of the mediation model.

### Mediation analysis: discrimination as a mediator between Black Catholic identity and mental health

We conducted a mediation analysis using the PROCESS macro to examine whether discrimination (M: D_Total) mediated the relationship between Black Catholic identity (X: C_BAfr) and mental health (Y: Mental Component Score, MCS). Model 4 was specified, with Black Catholic identity as the independent variable, discrimination as the mediator, and MCS as the outcome variable.

A subset of Black Catholics was analyzed from a larger dataset (*N* = 285). Bootstrapping with 5,000 resamples was used to generate bias-corrected confidence intervals (CIs) for indirect effects. The analysis assessed both the direct effect of Black Catholic identity on MCS and the indirect effect via discrimination.

Standardized regression coefficients (*b*), standard errors (*SE*), and 95% confidence intervals were reported for both direct and indirect effects. Model fit was evaluated using R² and F-statistics to determine the proportion of variance explained in the outcome variable.

### Mediation analysis: patient satisfaction as a mediator between financial stress and mental health

A mediation analysis was conducted using the PROCESS macro (Model 4) in SPSS to investigate the relationship between financial stress (raw_fin), patient satisfaction (MDSTR_R), and Mental Component Summary score (MCS). The sample consisted of 285 participants. Mental Component Summary score (MCS) was used as the dependent variable, financial stress (raw_fin) as the independent variable, and patient satisfaction (MDSTR_R) as the mediator.

The PROCESS macro employed 5,000 bootstrap samples to compute confidence intervals for the indirect effects of financial stress on Mental Component Summary score via patient satisfaction. Statistical significance was evaluated using a 95% confidence interval. The results were analyzed to determine the direct and indirect effects, assessing whether patient satisfaction mediated the relationship between financial stress and mental well-being.

### Latent class analysis

Latent Class Analysis was performed using Mplus 8.6 (Muthén & Muthén [77], Los Angeles, CA) (Muthen_Muthen_MPLUS) to identify distinct profiles based on Black Catholic respondents’ stress timelines and financial stress levels. The analysis utilized four main indicators: the timeline for financial stress (chronic, life trauma, daily hassle, or acute), the Financial Stress Scale (for quantifying financial stress), the mental component score (for determining mental wellness) and a single-item question assessing stress level, aligned with the social-ecological model, and Black Catholic identity, which was explicitly included as a latent class indicator. For model fit, we evaluated classes ranging from 2 to 6 and used criteria such as Akaike Information Criterion (AIC), Bayesian Information Criterion (BIC), Entropy, Lo-Mendell-Rubin Adjusted Likelihood Ratio Test (LMR), and the Bootstrapped Likelihood Ratio Test (BLRT). Lower AIC and BIC values, higher Entropy, and significant LMR and BLRT values guided model selection to ensure the best fit for the data.

### Structural equation modeling

We applied structural equation modeling using Mplus to evaluate our hypothesized framework. SEM was chosen due to its capacity to integrate both measurement and structural models, allowing us to visualize latent constructs and assess direct and indirect relationships among variables. This approach was especially suited to examining the multi-layered interactions outlined in our model, capturing the complexity of social-ecological factors and the need to capture the relationship between variables and their modifiers as it related to chronic stress-related risk.

The SEM analysis included three main areas of focus: timelines (representing stress duration), measures (financial stress and mental component scores), and moderation (including discrimination, patient satisfaction and religious affiliation). PROCESS macro and regression analysis in SPSS complemented this model to explore specific moderating effects. Model fit was assessed using a range of indices. The chi-square test provided an initial fit measure, with a non-significant p-value (p > 0.05) and a chi-square/df ratio of < 3 indicating an acceptable fit. Additional fit indices included the Comparative Fit Index (CFI > 0.90) and the Tucker-Lewis Index (TLI > 0.90). The Root Mean Square Error of Approximation (RMSEA) was also evaluated, with values < 0.05 considered indicative of good fit, alongside the Standardized Root Mean Square Residual (SRMR) measure, where lower values denote better fit.

### Structural equation models comparing financial stress and perceived stress score as predictors of chronic risk

This study employed a structural equation modeling (SEM) approach to examine the relationship between financial stress, perceived stress, discrimination, and chronic risk among Black Catholics classified as having chronic stress (Profile 1). The analysis was conducted using data from a sample of Black Catholics, with model comparisons focused on different predictors of chronic stress outcomes.

#### Measures.

The study included multiple measures to assess stress and its impact on chronic risk. Financial stress (MP_FIN) was measured as a continuous variable assessing self-reported financial strain. To examine the potential differences between financial stress and general stress, the Perceived Stress Scale (PSSNEW) was included as an alternative measure of perceived stress. Discrimination (D_TOTAL) was also measured as a predictor, given its potential influence on chronic stress experiences.

The Black Catholic Profile (MPRO_1BC) was modeled as a latent variable, identifying Black Catholics who exhibited chronic stress characteristics based on a latent class analysis. The outcome variable, chronic risk (CHRONRSK), was treated as a dichotomous measure to classify individuals who self-reported persistent financial stress.

#### Analytical approach.

Structural equation modeling (SEM) was conducted using Mplus with maximum likelihood estimation (ML). Two models were specified to compare the effects of financial stress vs. perceived stress score on chronic risk:

**Model 1** (Financial Stress Model): Examined the relationships between financial stress, discrimination, and chronic risk, with the Black Catholic profile as a mediating variable.

**Model 2** (Perceived Stress Model): Substituted the Perceived Stress Scale (PSSNEW) in place of financial stress to determine whether a Perceived Stress Scale measure could serve as an alternative predictor.

Model fit was assessed using established goodness-of-fit indices, including the Root Mean Square Error of Approximation (RMSEA), Comparative Fit Index (CFI), Tucker-Lewis Index (TLI), and Standardized Root Mean Square Residual (SRMR). Model comparisons were conducted to evaluate whether financial stress or the Perceived Stress Scale measures provided a better representation of chronic risk within this population.

#### Statement.

During the preparation of this work the author used OpenAI Large Language Model for draft refinement and summarization assistance. After using this tool/service, the author reviewed and edited the content as needed and take full responsibility for the content of the publication.

## Results

### Descriptive analysis: MCS distribution among low-income Black Catholics

**Fig 1:**
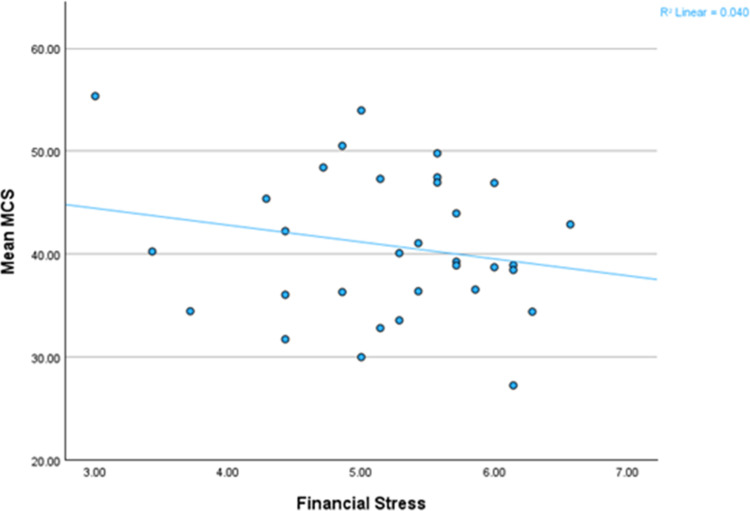
Impact of financial stress on MCS on Black Catholics.

Our study’s descriptive statistics indicated that, 90.6% of the 32 low-income Black Catholics assessed scored below the national average on the MCS [75] Conversely, 20.7% of all the participants (N = 285) achieved or surpassed this benchmark. This distribution is depicted visually in Fig 1, which demonstrates the influence of financial stress on MCS among Black Catholics.

#### Model fit and comparison: financial stress vs. perceived stress.

**Fig 2:**
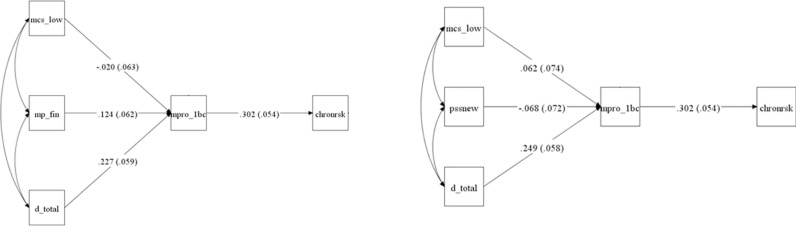
Comparison of structural equation models using perceived stress vs. financial stress as predictors (both models include discrimination and MCS). Notes: Path coefficients are standardized (STDYX); standard errors are shown in parentheses. All models were estimated with structural equation modeling in Mplus. mp_fin = financial stress. d_total = discrimination. mpro_1bc = Black Catholic profile (profile 1 = “chronic” from the latent Class Analysis). chronrsk = they self-selected chronic from the timeline of their financial stress. mcs_low = low mental component score. pssnew = Perceived Stress Scale.

To determine the most appropriate predictor chronic financial-stress risk in Black populations, as shown in Fig 2, we conducted structural equation modeling analyses comparing models that included financial stress (MP_FIN) and general perceived stress (PSSNEW) as predictors.

### Model fit indices

The financial stress model demonstrated superior fit compared to the Perceived Stress Scale model. The chi-square goodness-of-fit test for the financial stress model was χ²(3) = 3.978, p = 0.2639, whereas the Perceived Stress Scale model produced χ²(3) = 17.678, p = 0.0005, suggesting poor fit. Additional fit indices further confirmed these differences:

**Financial stress model:**

CFI/TLI: 0.979/ 0.952
RMSEA: 0.034 (90% CI: 0.000–0.111)
SRMR: 0.037
AIC/BIC: 121.793/ 151.013

**Perceived stress model:**

CFI/TLI: 0.747/ 0.410
RMSEA: 0.131 (90% CI: 0.076–0.193)
SRMR: 0.075
AIC/BIC: 124.854/ 154.074

These findings indicate that financial stress is a stronger predictor within this model, as the Perceived Stress Scale model fails to meet conventional fit thresholds.

### Direct and indirect effects

#### Impact on the Black Catholic profile (MPRO_1BC).

In the financial stress model, financial stress (MP_FIN) significantly predicted the Black Catholic profile (MPRO_1BC) (β = 0.124, SE = 0.062, p = 0.045), while discrimination (D_TOTAL) was a stronger predictor (β = 0.227, SE = 0.059, p < 0.001).

In the perceived stress model, the Perceived Stress Scale (PSSNEW) did not significantly predict MPRO_1BC (β = -0.068, SE = 0.072, p = 0.349), indicating that the Perceived Stress Scale does not contribute meaningfully to this framework.

#### Impact on chronic risk (CHRONRSK).

The financial stress model demonstrated that MPRO_1BC significantly predicted chronic risk (CHRONRSK) (β = 0.302, SE = 0.054, p < 0.001).

In the perceived stress model, the same effect was present (β = 0.302, SE = 0.054, p < 0.001), but the poor fit of the model suggests that this pathway is less meaningful when the Perceived Stress Scale is included as a predictor.

#### R-square values.

The financial stress model accounted for more variance in chronic risk and the Black Catholic profile:

**Financial stress model:**

CHRONRSK R² = 0.091 (SE = 0.033, p = 0.005)
MPRO_1BC R² = 0.078 (SE = 0.030, p = 0.011)

**Perceived stress model:**

CHRONRSK R² = 0.091 (SE = 0.033, p = 0.005)
MPRO_1BC R² = 0.066 (SE = 0.028, p = 0.021)

### Frequency analysis

The findings of a frequency analysis revealed a disproportionate representation of Black Catholics in the sample when contrasted with national averages. According to national statistics, the proportion of Black Catholics in the population is 4% [53]. However, in our sample, Black Catholics constituted 11.23% of the participants. Furthermore, Black Protestants were found to be underrepresented.

### Demographic composition

Table 1 provides a demographic breakdown of participants by racial group and religious affiliation.

**Table 1:** Demographic comparison of religious affiliations by race.

Demographic Variable	Non-Black N (%)	Black Protestant N (%)	Black Catholic N (%)	Black Atheist/ Agnostic N (%)	Black Nothing in Particular N (%)	P Value
Income Status						0.001
Less than $21,960	137 (62.6%)	14 (48.3%)	3 (9.4%)	2 (66.7%)	2 (100.0%)	
$21,961 to $35,580	48 (21.9%)	6 (20.7%)	13 (40.6%)	1 (33.3%)	0 (0.0%)	
$35,581 to $53,740	34 (15.5%)	9 (31.0%)	16 (50.0%)	0 (0.0%)	0 (0.0%)	
Education Status						0.001
Less than high school	4 (1.8%)	0 (0.0%)	1 (3.1%)	0 (0.0%)	0 (0.0%)	
High school diploma	24 (11.0%)	3 (10.3%)	1 (3.1%)	0 (0.0%)	0 (0.0%)	
No degree	86 (39.3%)	12 (41.4%)	1 (3.1%)	2 (66.7%)	2 (100.0%)	
Bachelor’s degree	80 (36.5%)	10 (34.5%)	14 (43.8%)	1 (33.3%)	0 (0.0%)	
Master’s degree	25 (11.4%)	4 (13.8%)	15 (46.9%)	0 (0.0%)	0 (0.0%)	
Relationship Status						0.001
Married	87 (39.7%)	9 (31.0%)	30 (93.8%)	1 (33.3%)	0 (0.0%)	
Divorced	15 (6.8%)	0 (0.0%)	0 (0.0%)	0 (0.0%)	0 (0.0%)	
Separated	7 (3.2%)	0 (0.0%)	0 (0.0%)	0 (0.0%)	0 (0.0%)	
Widowed	7 (3.2%)	0 (0.0%)	0 (0.0%)	0 (0.0%)	0 (0.0%)	
Unmarried	103 (47.0%)	20 (69.0%)	2 (6.3%)	2 (66.7%)	2 (100.0%)	
Employment Status						0.001
Full-time employment	100 (45.7%)	14 (48.3%)	31 (96.9%)	1 (33.3%)	1 (50.0%)	
Part-time employment	29 (13.2%)	7 (24.1%)	0 (0.0%)	0 (0.0%)	0 (0.0%)	
Unemployed	32 (14.6%)	5 (17.2%)	0 (0.0%)	0 (0.0%)	1 (50.0%)	
Self-employed	33 (15.1%)	2 (6.9%)	1 (3.1%)	1 (33.3%)	0 (0.0%)	
Home-maker	10 (4.6%)	0 (0.0%)	0 (0.0%)	0 (0.0%)	0 (0.0%)	
Student	7 (3.2%)	1 (3.4%)	0 (0.0%)	1 (33.3%)	0 (0.0%)	
Retired	8 (3.7%)	0 (0.0%)	0 (0.0%)	0 (0.0%)	0 (0.0%)	

### Stress profiles and health outcomes among Black Catholics

The descriptive statistics for sociodemographic variables in Table 2, across the three profiles of Black Catholics show distinct patterns in financial stress (FinSTRav), Mental Component Summary scores (MCS), Physical Component Summary scores (PCS), and discrimination levels (D_Total Discrimination). Profile 1, which reported the highest financial stress (mean = 5.79), also showed the lowest mental component score (MCS mean = 39.35) and Physical Component Summary score (PCS mean = 35.45). In contrast, Profile 3 exhibited the lowest financial stress (mean = 4.67) and the highest MCS (mean = 42.31) and PCS (mean = 43.05), indicating a distinct health profile compared to Profile 1. Additionally, discrimination levels varied, with Profile 1 reporting the highest average score (6.33 ± 2.40) and Profile 3 reporting the lowest (2.86 ± 2.12).

**Table 2:** Descriptive statistics of sociodemographic variables for Black Catholics by profile.

Variable	Profile 1 (N = 9)	Profile 2 (N = 16)	Profile 3 (N = 7)
FinSTRav	5.79	5.13	4.67
MdSTR_rev	3.92	4.36	4.55
MCS	39.35	40.97	42.31
PCS	35.45	38.70	43.05
D_Total Discrimination	6.33	5.44	2.86

Notes: FinSTRav, financial stress; MdSTR_rev, patient satisfaction; MCS, mental component score; PCS, physical component score; D_Total discrimination

### Financial stress timelines and health measures by profile

A chi-square analysis was conducted to examine the relationship between financial stress impact levels and latent profiles for Black Catholics. As shown in the Table 3, distinct patterns of financial stress emerged across profiles. In Profile 1, 28.1% of individuals reported financial stress as a chronic issue, while no respondents in Profiles 2 or 3 selected this timeline. Profile 2 exhibited two types of financial stress impacts, with 34.4% indicating a financial crisis affecting life and 15.6% identifying an unexpected financial event threatening life. Finally, Profile 3 primarily associated financial stress with daily hassles, with 21.9% reporting this timeline. The analysis showed statistically significant differences in stress impact timelines between profiles (p < .001).

**Table 3:** Impact of financial stress on life by profile for Black Catholics with chi-square analysis^†^.

Category	Profile 1 (Count, %)	Profile 2 (Count, %)	Profile 3 (Count, %)
Chronic Finance	9, 28.1%	0, 0.0%	0, 0.0%
Finance Life Event Effect Life	0, 0.0%	11, 34.4%	0, 0.0%
Unexpected Finances Threatened Life	0, 0.0%	5, 15.6%	0, 0.0%
Finances Daily Hassle	0, 0.0%	0, 0.0%	7, 21.9%

Chi-square test results  : p = < .001

†More than 20% of expected cells were <5; χ² descriptive only.

### Structural equation model: pathways linking financial stress, discrimination, and mental health to chronic risk via the Black Catholic profile

**Fig 3:**
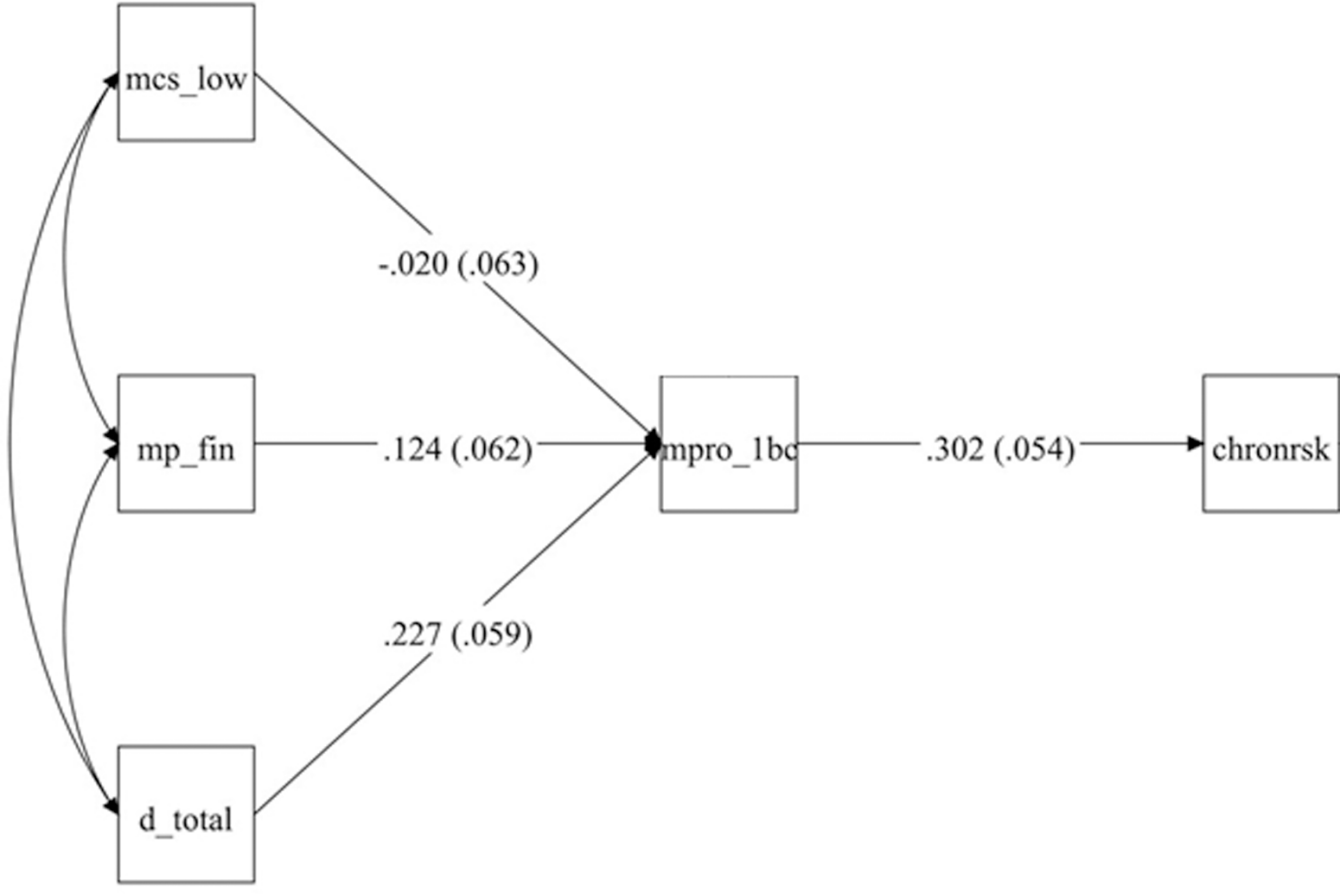
Structural equation model illustrating the impact of financial stress and discrimination on mental component summary score and chronic stress among Black Catholics. Notes: Path coefficients are standardized (STDYX); standard errors are shown in parentheses. All models were estimated with structural equation modeling in Mplus. **mp_fin = financial stress. d_total = discrimination. mpro_1bc = Black Catholic profile (profile 1 = “chronic” from the latent class analysis). chronrsk = they self-selected chronic from the timeline of their financial stress.** mcs_low = low Mental Component Summary score.

The structural equation model shown in Fig 3 examines the relationship between financial stress (MP_FIN), discrimination (D_Total), mental component score (MCS_LOW), and chronic stress (CHRONRSK) which equates as our proxy for chronic financial-stress risk, through the Black Catholic profile (MPRO_1BC), demonstrating a good fit to the data. Key model fit indices indicated that the model adequately represented the observed relationships among variables: χ2(3)=3.978,p = 0.2639, RMSEA = 0.034 with a 90% confidence interval [0.000, 0.111], probability RMSEA ≤ 0.05 = 0.534, CFI = 0.979, TLI = 0.952, and SRMR = 0.037. These fit indices suggest that the hypothesized relationships among variables provide a close fit to the observed data.

### Path coefficients and direct effects

The standardized path coefficients (STDYX) are as follows:

**Direct effects**

MPRO_1BC on CHRONRSK: The Black Catholic profile (MPRO_1BC) has a significant positive effect on chronic stress (CHRONRSK) (β = 0.302, SE = 0.054, p < 0.001), suggesting that an increase in characteristics of the Black Catholic profile directly predicts an increase in chronic stress levels.
Financial Stress (MP_FIN) on MPRO_1BC: Financial stress significantly influences the Black Catholic profile (β = 0.124, SE = 0.062, p = 0.047), indicating that higher levels of financial stress contribute to an increase in the Black Catholic profile characteristics.
MCS_LOW on MPRO_1BC: The mental component score (MCS_LOW) does not have a significant effect on the Black Catholic profile (β=−0.020, SE = 0.063, p = 0.746), suggesting that MCS does not significantly contribute to this profile when financial stress is included in the model.
D_Total on MPRO_1BC: D_Total, another predictor, shows a positive and significant effect on MPRO_1BC (β = 0.227, SE = 0.059, p < 0.001), contributing substantially to the Black Catholic profile.

**Mediating factors and indirect effects**

- MPRO_1BC as a Mediator: The Black Catholic profile (MPRO_1BC) acts as a significant mediator in the pathway from financial stress (MP_FIN) to chronic stress (CHRONRSK). The indirect effect of financial stress on chronic stress through the Black Catholic profile suggests that financial stress indirectly increases chronic stress levels by first enhancing characteristics associated with the Black Catholic profile. This mediating pathway was statistically significant (p < 0.05 for MP_FIN’s effect on MPRO_1BC and MPRO_1BC’s effect on CHRONRSK).
- Non-Significance of MCS_LOW: Despite initial hypotheses, the Mental Component Summary score (MCS_LOW) did not significantly contribute to the Black Catholic profile or chronic stress. Consequently, it does not act as a mediator in this model, and its inclusion did not impact the mediation effect on chronic risk.

### Structural equation model: financial stress and discrimination as predictors of chronic risk via the Black Catholic profile

**Fig 4:**
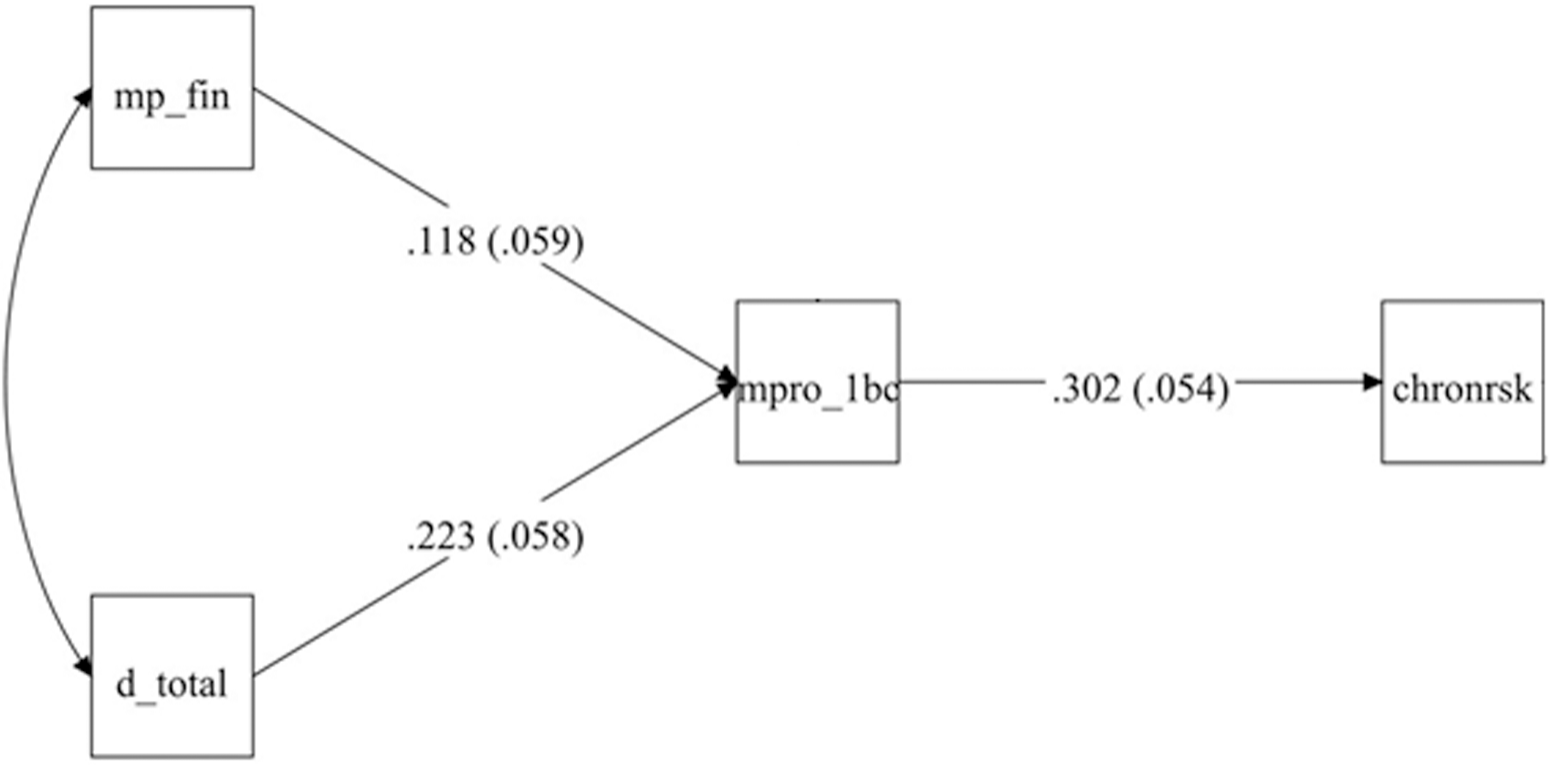
Structural equation model of the impact of financial stress and discrimination on chronic stress among Black Catholics. Notes: Path coefficients are standardized (STDYX); standard errors are shown in parentheses. All models were estimated with structural equation modeling in Mplus. mp_fin = financial stress. d_total = discrimination. mpro_1bc = Black Catholic Profile (profile 1 = “chronic” from the latent class analysis). chronrsk = they self-selected chronic from the timeline of their financial stress.

The structural equation model shown in Fig 4 examines the relationship between financial stress (MP_FIN), discrimination (D_Total), and chronic stress (CHRONRSK), our proxy for chronic financial-stress risk, through the Black Catholic profile (MPRO_1BC), demonstrating a good fit to the data:

Chi-Square Goodness-of-Fit Test: χ2(2)=1.720,p=0.4232
RMSEA: Estimate = 0.000, 90% CI [0.000, 0.112], with Probability RMSEA ≤ 0.05 = 0.635
CFI/TLI: CFI = 1.000, TLI = 1.000
SRMR: 0.024

These fit indices are markedly improved compared to the model incorporating both financial stress and MCS. The lower Akaike (AIC = 119.898) and Bayesian (BIC = 145.465) information criteria scores also suggest that the simpler model provides a better fit to the data, underscoring the strength of financial stress as the primary predictor in this context.

Path coefficients and direct effects

**The standardized path coefficients (STDYX) reveal significant direct effects as follows:**

Direct effects
Financial Stress (MP_FIN) on MPRO_1BC: Financial stress has a significant positive effect on the Black Catholic profile (β = 0.118, SE = 0.059, p = 0.045). This indicates that higher levels of financial stress contribute to the characteristics associated with the Black Catholic profile.
Discrimination (D_TOTAL) on MPRO_1BC: The total discrimination score also has a significant positive effect on the Black Catholic profile (β = 0.223, SE = 0.058, p < 0.001), further influencing this profile.
MPRO_1BC on CHRONRSK: The Black Catholic profile is a significant predictor of chronic stress (β = 0.302, SE = 0.054, p < 0.001), suggesting that individuals with characteristics of this profile are at greater risk for chronic stress.
Mediating role of MPRO_1BC

**Indirect effects and mediation:**

The Black Catholic profile (MPRO_1BC) serves as a mediator in the relationship between financial stress (MP_FIN) and chronic stress (CHRONRSK). Financial stress indirectly contributes to chronic stress by first impacting the Black Catholic profile, which in turn elevates chronic stress levels. This mediation effect suggests that the Black Catholic profile plays a critical role in translating financial stress into chronic stress.

**Indirect effect significance:**

The pathway from financial stress through the Black Catholic profile to chronic stress is statistically significant. The direct path from financial stress to chronic stress is not modeled explicitly here, reinforcing the role of the Black Catholic profile as a central mediator. This mediation highlights the pathway through which financial stress impacts chronic stress, underscoring the influence of socio-demographic factors captured in the Black Catholic profile.

### Mediation of Black Catholic identity and mental health via financial stress

The mediation analysis in Table 4 revealed a significant **direct effect** of Black Catholic status on Mental Component Summary score (MCS) (b = 4.09, p = 0.0288, 95% CI [0.4260, 7.7527]), indicating that being a Black Catholic is associated with an increase in MCS scores when controlling for financial stress. However, financial stress significantly mediated this relationship. The indirect effect of Black Catholic status on MCS through financial stress was negative and significant (b=−3.94, 95% CI [-5.5815, -2.4389]), suggesting that higher financial stress diminishes the positive association between Black Catholic status and mental well-being.

**Table 4:** Mediation of Black Catholic status and MCS by financial stress.

Path	Coefficient	SE	t	p	95% CI for Indirect Effect
X to M (a-path)	7.3814	2.0130	3.6669	.0003	
M to Y (b-path)	−0.5334	0.0537	−9.9331	<.0001	
X to Y (c-path)	4.0893	1.8611	2.1973	.0288	
Indirect Effect (Financial_Stress)	−3.9370	0.7962			[−5.5815, −2.4389]
Total Effect (c-path)					

Notes:

X = Black_Catholic (independent variable).

M = Financial_Stress (mediator).

Y = MCS (dependent variable).

The indirect effect is computed as the product of the a−path and b−path coefficients (a*b = 7.3814 * −0.5334 = −3.9370).

Confidence intervals for the indirect effect are bias-corrected bootstrap confidence intervals.

If the confidence interval for the indirect effect does not include zero, as in this case, the indirect effect is considered statistically significant.

The overall model explained **25.92%** of the variance in MCS scores (R2 = 0.2592, F(2, 282) = 49.34, p < 0.001). Financial stress had a strong negative impact on MCS (b=−0.53, p < 0.001, indicating that higher financial stress was associated with lower Mental Component Summary scores among Black Catholics.

### Paradox in health outcomes

Our research findings in Table 5 showed that Black Catholics recorded the lowest average PCS and MCS scores **versus White Catholics**, indicating poorer physical and mental health outcomes. Moreover, Black Catholics reported the highest levels of financial stress (36.50) and discrimination (5.12), highlighting the compounded effect of these stressors on their health. It is worth noting that Black Atheists/Agnostics exhibited an inverse pattern, with the highest PCS scores (52.61) and the lowest MCS scores (29.03). In comparison to the broad White category, White Catholics, despite higher reported levels of discrimination and financial stress, had lower PCS scores (40.54) and higher MCS (41.97) scores.

**Table 5:** Analysis of health outcomes by religious affiliation.

	MCS (SD)	PCS (SD)	Financial Stress (SD)	Discrimination (SD)	*N*
Black Catholic	40.81 (6.96)	38.74 (6.63)	36.50 (5.91)	5.13 (3.17)	32
Black Protestant	40.91 (9.72)	42.99 (10.88)	31.38 (11.07)	2.83 (2.45)	29
Black Atheist/ Agnostic	29.04 (7.96)	52.62 (9.87)	28.00 (8.19)	1.00 (1.00)	3
Black Nothing in Particular	50.45 (9.72)	45.53 (12.33)	18.00 (2.83)	1.50 (0.71)	2
White Catholic	41.98 (9.29)	40.55 (7.08)	32.41 (9.34)	2.90 (2.65)	49
Hispanic Catholic	41.02 (5.18)	45.65 (8.81)	26.00 (9.90)	1.00 (1.41)	2
All Blacks	40.70 (8.76)	41.50 (9.46)	32.86 (9.45)	3.90 (3.07)	71
All White	41.09 (11.63)	44.20 (9.59)	29.03 (11.19)	2.30 (2.44)	181

### LCA Profile Selection

Table 6 presents the model fit statistics for different latent class models, revealing that the 3-class solution was optimal. The 3-class model displayed lower AIC (1682.636) and BIC (1777.600) values compared to the 2-class model, and its ** overall interpretability** exceeded those of models with more classes (4–6). The LMR and BLRT p-values for the 4-class solution and beyond exceeded the threshold of.05, indicating less reliable fit than the 3-class model. The final 3-class solution provided interpretable profiles aligned with Black Catholic responses and their stress timeline selections, making it the most optimal solution.

**Table 6:** Latent class analysis fit statistics for 2–6 class models.

Number of Classes	AIC	BIC	Entropy	LMR	BLRT
2-class	1763.106	1825.198	1.000	0.0000	0.0000
3-class	1682.636	1777.600	1.000	0.0000	0.0000
4-class	1656.000	1783.837	0.976	0.0513	0.0538
5-class	1672.864	1833.574	0.831	0.6423	0.6439
6-class	1690.864	1884.446	0.861	0.5894	0.5894

Notes: AIC, Akaike information criterion; BIC, Bayesian information criterion; LMR, Lo–Mendell–Rubin adjusted likelihood ratio test p-value; BLRT, bootstrap likelihood ratio test p-value.

**Fig 5:**
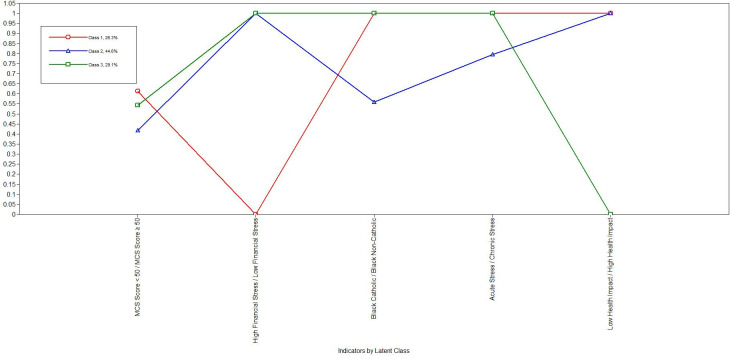
Conditional probabilities of stress indicators across three profiles among Black Catholics.

Fig 5 illustrates the conditional probabilities for each indicator across the three identified profiles: Profile 1, Profile 2, and Profile 3. Profile 1 includes Black Catholics who primarily reported chronic financial stress. Profile 2 represents those indicating life trauma as the timeline for their financial stress experiences. Profile 3 comprises respondents identifying daily financial stress as their main timeline for stress exposure.

#### Analysis of variance findings for Black Catholics with various religions.

A two-way ANOVA was conducted to examine the impact of financial stress and religious affiliation on Mental Component Summary scores (MCS) among participants from various religious backgrounds **(N = 259)**. The overall model was significant (F (16, 242) = 3.914, p < .001), explaining 15.3% of the variance in MCS scores (Adjusted R² = .153). Financial stress had a significant effect on MCS (F (2, 242) = 7.533, p < .001), with higher financial stress being associated with lower mental well-being. Additionally, religious affiliation significantly affected MCS (F (2, 242) = 3.379, p = .036), but there were no significant differences between Black Catholics and other religious groups, including Black Protestants (F (2, 242) =.100, p = .905). The interaction between financial stress and religious affiliation was not significant, indicating that the effect of financial stress on Mental Component Summary score did not differ significantly between religious groups. Post-hoc comparisons revealed that individuals in the low financial stress group had significantly higher MCS scores than those in the medium and high stress groups.

### ANCOVA: contextual stressors and MCS in Black religious groups

A one-way analysis of covariance (ANCOVA) was conducted to examine the effects of neighborhood stress, social support, patient satisfaction, and discrimination on MCS among Black individuals from different religious affiliations. The analysis presented in Table 7, focused exclusively on Black participants, including those identifying as Black Protestant, Black Catholic, Black Atheist/Agnostic, and Black Nothing in Particular.

**Table 7:** Additional influencing factors on mental component summary score (MCS) among Black religious groups.

Source	df	Mean Square	Sig.
NeSTR_av	1	41.451	.416
SoSTR_av	1	120.432	.166
MdSTR_rev	1	13752.528	<.001
D_Total	1	24.071	.535

Notes:

NeSTR_av = Neighborhood Stress

SoSTR_av = Social Support

MdSTR_rev = Patient satisfaction

D_Total = Discrimination

The overall model was statistically significant, F (8, 276) = 37.114, p < .001, explaining 50.4% of the variance in MCS scores (Adjusted R² = .504). While financial stress had already been established as a significant factor in prior analyses, this ANCOVA was conducted to assess the influence of additional variables. Among the covariates, patient satisfaction (MdSTR_rev) emerged as the only significant predictor of MCS, F (1, 276) = 220.358, p < .001, indicating that lower satisfaction with medical care was strongly associated with poorer mental well-being. Other factors, including neighborhood stress (F (1, 276) = 0.664, p = .416), social support (F (1, 276) = 1.930, p = .166), and discrimination (F (1, 276) = 0.386, p = .535), were not significant predictors of MCS. Additionally, religious affiliation did not have a significant effect on MCS, F (4, 276) = 1.403, p = .233. Unemployment was not analyzed, as 100% of Black Catholics in the sample were employed.

## Patient satisfaction

### One-way ANOVA: patient satisfaction by MCS group

A one-way ANOVA was conducted to examine the differences in the patient satisfaction (MDSTR_R) between individuals with below-average and above-average Mental Component Summary scores. The analysis revealed a statistically significant difference in patient satisfaction between the two groups, F (1, 283) = 153.625, p < .001, with a large effect size (Eta-squared = 0.352). Participants with below-average MCS scores reported significantly more negative perceptions of their medical care (M = 1.84, SD = 0.59) compared to those with above-average MCS scores (M = 0.78, SD = 0.54). This suggests that individuals with poorer mental health tend to perceive their medical care more negatively. Levene’s test for homogeneity of variances confirmed that the assumption of equal variances was met (p > .05).

### Mediation analysis results using process model 4 for financial stress, patient satisfaction and MCS

A mediation analysis, as shown in Table 8, using the PROCESS macro (Model 4) was conducted to examine whether the patient satisfaction (MDSTR_R) mediates the relationship between financial stress (raw_fin) and Mental Component Summary score (MCS).model was significant (F(2, 282) = 147.77, p < .001), explaining 51.17% of the variance in MCS scores (R² = .5117).The results show that financial stress (raw_fin) was a significant predictor of the patient satisfaction (MDSTR_R) (B = 0.0391, p < .001), indicating that greater financial stress was associated with poorer patient satisfaction. Additionally, patient satisfaction was found to have a significant negative effect on MCS scores (B = -9.9310, p < .001), suggesting that worse patient satisfaction is strongly linked to lower mental well-being.

**Table 8:** Mediation analysis results using PROCESS model 4 for financial stress, patient satisfaction, and MCS.

Path	Coefficient	SE	t	p	95% CI for Indirect Effect
X to M (a-path)	0.0391	0.0032	12.3978	<.001	
M to Y (b-path)	-9.931	0.8024	-12.376	<.001	
X to Y (c-path)	-0.1198	0.0529	-2.2633	.0244	
Indirect Effect (Financial_Stress)	-0.3885	0.0458			[-0.4832, -0.3018]
Total Effect (c-path)					

Notes:

• X = Financial Stress (independent variable).

• M = Patient satisfaction (mediator).

• Y = MCS (dependent variable).

• The indirect effect is computed as the product of the a−path and b−path coefficients.

• Confidence intervals for the indirect effect are bias-corrected bootstrap confidence intervals.

• If the confidence interval for the indirect effect does not include zero, as in this case, the indirect effect is considered statistically significant.

The direct effect of financial stress on MCS was also significant (B = -0.1198, p = .0244), indicating that financial stress negatively impacts mental health. However, the indirect effect of financial stress on MCS through patient satisfaction was larger and significant (indirect effect = -0.3885, 95% CI [-0.4832, -0.3018]), suggesting that a considerable portion of the effect of financial stress on Mental Component Summary score is mediated by patient satisfaction

### Chi-square analysis: association between financial stress levels and perceived sources of stress

A chi-square analysis, as shown in Table 9, was conducted to examine the relationship between financial stress levels and the perceived sources of financial stress across multiple social-ecological levels (personal, relational, institutional, community, and policy). The results indicated a significant association between financial stress and the level at which stress is perceived (χ²(4) = 13.526, p = 0.009), suggesting that individuals experiencing high financial stress differ systematically in how they attribute stress sources.

**Table 9:** Chi-square analysis of financial stress and areas of concern^†^.

Financial Stress Level	Personal Level	Relationship Level	Institutional Level	Community Level	Policies, Laws, and Regulations	Total
Low Stress (Quartile 1 & 2)	0	3	1	1	2	7
% Within Stress Level	0.0%	42.9%	14.3%	14.3%	28.6%	100.0%
% of Total	0.0%	9.4%	3.1%	3.1%	6.3%	21.9%
High Stress (Quartile 3 & 4)	10	11	4	0	0	25
% Within Stress Level	40.0%	44.0%	16.0%	0.0%	0.0%	100.0%
% of Total	31.3%	34.4%	12.5%	0.0%	0.0%	78.1%
Total	10	14	5	1	2	32
% Within Stress Level	31.3%	43.8%	15.6%	3.1%	6.3%	100.0%
% of Total	31.3%	43.8%	15.6%	3.1%	6.3%	100.0%

† 80% of expected cells <5; χ^2^ results are descriptive only.

### Financial stress and perceived stressors

Among individuals classified as experiencing low financial stress (Quartiles 1 & 2), the most frequently reported sources of stress were relationship-level (42.9%) and policy-level (28.6%) factors, followed by institutional (14.3%) and community (14.3%) stressors. No participants in this group identified personal-level financial stress as their primary concern.

Conversely, individuals in the high financial stress group (Quartiles 3 & 4) overwhelmingly identified personal (40.0%), relational (44.0%), and institutional (16.0%) stressors as their primary sources of financial distress. No participants in this group attributed financial stress to community-level or policy-level factors.

### Correlation analyses

To further examine the relationship between financial stress levels and perceived stressor attribution, Pearson’s correlation analysis demonstrated a strong negative association (r = -0.589, p < 0.001) between financial stress and the identification of policy and systemic stressors. Similarly, Spearman’s rank correlation (r = -0.497, p = 0.004) reinforced the pattern that higher financial stress was associated with an increased focus on personal and relational stress sources, rather than broader systemic determinants.

### Summary of findings

High financial stress individuals (Quartiles 3 & 4) were significantly more likely to attribute their financial difficulties to personal, relational, and institutional sources and did not identify policy or community-level factors as primary stressors.

Low financial stress individuals (Quartiles 1 & 2) were more likely to perceive systemic (policy, community-level) stressors as contributing to their financial challenges.

Significant negative correlations suggest that as financial stress increases, individuals’ perception of systemic stressors decreases, narrowing their focus to immediate, interpersonal stressors.

These results quantify the structural impact of financial stress on stress attribution and provide statistical support for the broader theoretical argument that financial stress constricts systemic awareness, reinforcing an individual-level focus on stress management.

### ANOVA: financial stress and mental health.

A one-way analysis of variance (ANOVA) was conducted to examine the relationship between financial stress and mental health (MCS scores) among Black individuals. The results indicated a statistically significant effect of financial stress on MCS scores, F (42, 242) = 5.51, p < .001. However, the effect size was moderate to high, with η² = .489, suggesting that financial stress accounted for 48.9% of the variance in MCS scores. Despite the statistical significance, the relatively low F-statistic suggests that the effect may vary across subgroups, warranting further investigation into how financial stress interacts with other demographic and contextual factors within this population.

### Correlation: financial stress and mental health

A Pearson correlation analysis was conducted to examine the relationship between financial stress and mental health among Black individuals, including Black Catholics. The analysis focused on the relevant subset of participants, with results indicating a significant negative correlation between financial stress (FinanceScore RAW) and Mental Component Summary score (MCS), *r* = -0.533, *p* < .001. This suggests that higher financial stress levels are associated with lower Mental Component Summary scores in this population.

### Perceived discrimination among Black religious group

A descriptive analysis was conducted to examine differences in perceived discrimination among Black individuals with different religious affiliations. The findings indicate substantial variation in mean discrimination scores, with Black Catholics reporting the highest levels of perceived discrimination (*M* = 5.13, *N* = 32).

This score is nearly twice as high as that of Black Protestants (*M* = 2.83, *N* = 29) and more than the general Black population baseline (*M* = 3.90, *N* = 71). Black Atheists/Agnostics and Black individuals with no religious affiliation reported the lowest perceived discrimination (*M* = 1.00, *N* = 3 and *M* = 1.50, *N* = 2, respectively).

These findings suggest that perceptions of discrimination differ notably based on religious affiliation, with Black Catholics experiencing disproportionately high levels of discrimination compared to other Black religious groups.

### Direct effects of discrimination on financial stress and Black Catholic profile

Regression analysis, as shown in Table 10, revealed that discrimination significantly predicted financial stress,

**Table 10:** mediation analysis results using PROCESS model 4 for the impact of discrimination on Black Catholic profile via financial stress.

Path	Coefficient	SE	t/Z-value	p-value	95% CI (LLCI, ULCI)
X to M (a-path) (Discrimination Financial Stress)	0.1939	0.0332	5.85	<.001	(0.1286, 0.2592)
M to Y (b-path) (Financial Stress Black Catholic Profile)	1.0028	0.4503	2.23	0.026	(0.1202, 1.8854)
X to Y (c’-path) (Discrimination Black Catholic Profile)	0.3601	0.1212	2.97	0.003	(0.1225, 0.5977)
Indirect Effect (X → M → Y)	0.1944	0.6677	—	—	(0.0889, 0.3867)

**Notes:**

**X = Discrimination Total (independent variable).**

**M = Financial Stress (mediator).**

**Y = Black Catholic Profile (dependent variable).**

The **indirect effect** is computed as the product of the a-path and b-path coefficients (**0.1939 × 1.0028 = 0.1944**).

Confidence intervals for the indirect effect are **bias-corrected bootstrap confidence intervals**.

Bootstrap results caution: The output notes a nonconvergence error during bootstrapping, meaning that the bootstrap confidence intervals should be interpreted with caution.

Log-odds metric: The logistic regression results are expressed in log-odds.

b = 0.1939, SE = 0.0332, t = 5.85, p <.001, 95% CI [0.1286, 0.2592].

This finding indicates that higher levels of discrimination were associated with increased financial stress among Black Catholics.

Logistic regression further demonstrated that financial stress was a significant predictor of the Black Catholic profile,

b = 1.0028, SE = 0.4503, Z = 2.23, p =.026, 95% CI [0.1202, 1.8854].

This suggests that individuals experiencing higher financial stress were significantly more likely to be classified in the high-risk cardiovascular disease profile.

Additionally, discrimination had a direct, significant effect on the Black Catholic profile,

b = 0.3601, SE = 0.1212, Z = 2.97, p =.003, 95% CI [0.1225, 0.5977].

This indicates that even after accounting for financial stress, discrimination remained a strong predictor of chronic risk in this population.

### Mediation effect of financial stress

The indirect effect of discrimination on the Black Catholic profile through financial stress was significant, indirect effect = 0.1944, BootSE = 0.6677, 95% BootCI [0.0889, 0.3867].

Since the confidence interval does not include zero, this confirms that financial stress significantly mediates the relationship between discrimination and chronic risk.

### Model fit indicators

Logistic regression model fit indices indicated a strong model:

**-2 Log-Likelihood** 58.74, **p < .001**
**McFadden’s R²** 0.2649
**Cox & Snell R²** 0.0716
**Nagelkerke R²** 0.2928

These values suggest that the inclusion of discrimination and financial stress significantly improved the model’s ability to predict chronic risk in Black Catholics.

### Process model 4: indirect effect of discrimination on MCS via Discrimination_Total

The mediation analysis detailed in Table 11 utilized the PROCESS Model 4 to investigate the influence of discrimination on MCS scores through Discrimination_Total as a mediator. The analysis identified significant paths from the independent variable to the mediator, from the mediator to the dependent variable, and a direct path from the independent variable to the dependent variable; however, the direct effect was not statistically significant. Despite this, a statistically significant indirect effect was observed, indicating the mediating role of Discrimination_Total in the relationship between discrimination and MCS scores among Black Catholics. Additionally, the analysis revealed an exacerbating effect of discrimination experiences on MCS score reductions, which contributed to 11.45% of the model variance. This insightful analysis is further complemented by mean financial stress scores across various religious affiliations and races, as presented in Table 12.

**Table 11:** Mediation analysis results using PROCESS model 4 for the impact of discrimination on MCS via Discrimination_Total.

Path	Coefficient	SE	t	p	95% CI for Indirect Effect
X to M (a-path)	2.8365	0.4689	6.0494	<.0001	
M to Y (b-path)	−0.7533	0.2641	−2.8530	0.0047	
X to Y (c’-path)	2.2891	2.2134	1.0342	0.3019	
Indirect Effect (Discrimination_Total)	−2.1368	0.7576			[−3.8501, −0.8976]
Total Effect (c-path)					

Notes:

X = Black_Catholic (independent variable).

M = Discrimination_Total (mediator).

Y = MCS (dependent variable).

The indirect effect is computed as the product of the a-path and b-path coefficients (a*b = 2.8365 * −0.7533 = −2.1368).

Confidence intervals for the indirect effect are bias-corrected bootstrap confidence intervals.

If the confidence interval for the indirect effect does not include zero, as in this case, the indirect effect is considered statistically significant.

**Table 12:** Analysis of mean financial stress scores across religious affiliations in a predominantly Black sample.

Religious Affiliation	Mean Financial Stress Score	Number of Respondents (*N*)
Other Affiliation	28.94	219
Black Protestant	31.38	29
Black Catholic	36.50	32
Black Atheist/Agnostic	28.00	3
Black Nothing in Particular	18.00	2
Total	29.95	285

### Moderation analysis: Catholic affiliation and financial stress on MCS

The moderation analysis shown in Table 13 indicated that Catholic affiliation significantly moderated the relationship between financial stress and MCS. The overall model was significant, F (3,281)=37.55, p < .0001, and explained 28.62% of the variance in MCS scores (R2 = 0.2862). Financial stress had a significant negative effect on MCS (B = −4.219, p < .0001), indicating that higher financial stress is associated with lower mental well-being.

**Table 13:** Moderation analysis: Catholic affiliation as a moderator of the relationship between financial stress and MCS.

Path	Coefficient	SE	t	p	95% CI
Constant	57.4211	1.7889	32.0979	0.0	[53.8996, 60.9425]
X (FinSTRav)	-4.219	0.4095	-10.3032	0.0	[-5.0251, -3.4130]
W (Catholic)	-5.8945	4.7085	-1.2519	0.2117	[-15.1629, 3.3739]
Interaction (X*W)	2.145	0.9659	2.2208	0.0272	[0.2437, 4.0463]

Notes:

X = FinSTRav (financial stress) (independent variable).

W = Catholic (moderator).

Y = MCS (dependent variable).

The interaction term (X*W) represents the moderation effect of Catholic on the relationship between Financial Stress and MCS.

Conditional effects show how the effect of Financial Stress on MCS varies at different levels of the moderator Catholic.

Confidence intervals for the effects are bias-corrected bootstrap confidence intervals.

While Catholic affiliation did not have a significant direct effect on MCS (B = −5.895, p = .2117), the interaction between financial stress and Catholic affiliation was significant (B = 2.145, p = .0272). This indicates that Catholic affiliation moderates the effect of financial stress on MCS, such that Catholics experience a less severe decline in Mental Component Summary score compared to non-Catholics when facing financial stress.

Specifically, for non-Catholics, the effect of financial stress on MCS was more pronounced (B=−4.219, p < .0001), while for Catholics, the negative effect was reduced (B = −2.074, p = .0184). This suggests that while financial stress has a detrimental effect on mental well-being, Catholic affiliation serves as a protective factor that mitigates some of the negative impact of financial stress.

## Discussion

### Chronic financial stress patterns and the BaCE pathway

Chronic financial stress has emerged as a critical yet profoundly underrecognized driver of cardiovascular disease among Black Catholics. Our findings clearly show a devastating reality: 90.6% of low-income Black Catholics scored below the national average on the Mental Component Summary scale [75] with financial stress alone accounting for nearly half (46%) of this severe mental health burden (η² = 0.489). This direct connection between chronic financial stress and declining mental health cannot be overstated, as it unequivocally positions these individuals on a path **associated with higher cardiometabolic vulnerability**. [8]. Yet, alarmingly, rather than being identified and actively integrated into established CVD screening and intervention protocols, this systematically marginalized group is left abandoned on a chronic stress trajectory—one that is neglected by current risk assessment and prevention frameworks [8]. Even more disturbingly, chronic stress itself is recognized not merely as a risk factor for illness but as a predictor of early mortality [78].

This systemic misclassification of chronic financial stress as a mere ancillary factor rather than as a central determinant of CVD health [14] represents a profound and morally unacceptable public health failure. The evidence is stark: chronic stress alone increases the occurrence of coronary heart disease by 40–50% [79]. Our research explicitly highlights that individuals facing the combined burden of intense financial stress and severely impaired mental health are not suffering isolated incidents of distress—they are embedded within persistent, systemic stress trajectories that actively drive CVD progression. This conclusion is supported unequivocally by Chida’s [36] meta-analysis, which confirms the harmful effects of acute mental stress reactivity on future cardiovascular outcomes, and advocates explicitly for stress management as an essential component of cardiovascular prevention. Yet, tragically and unjustifiably, these Black Catholic populations remain invisible within standard cardiovascular risk assessments, untracked and unsupported in existing clinical frameworks [8]. The implications of such systemic neglect go far beyond individual tragedy; they underscore profound structural racism in the identification and prevention of CVD among marginalized communities [3]. Lewis [80] explicitly identifies that a critical limitation in existing chronic stress research is the reliance on clinically manifested disease, which systematically overlooks early-stage disease development directly attributable to chronic stress—further compounding disparities in care.

The Black Cardiovascular Ecological Pathway explicitly addresses these systemic failures through a structured social-ecological approach designed to proactively identify, monitor, and intervene in financial stress at multiple social-ecological levels. Rather than trivializing financial stress as an isolated personal issue, the BaCE Pathway systematically exposes financial stressors, their pervasive patterns, and their direct influence on CVD risk among Black subpopulations. By mapping financial stress across various social-ecological contexts, we can identify precise intervention points that actively redirect affected individuals away from chronic stress pathways toward meaningful, life-saving preventive strategies. Such structured social-ecological frameworks have successfully mitigated cardiovascular risk factors globally for over four decades [23].

This research highlights the absolute urgency of early identification, rigorous pattern recognition, and targeted interventions to disrupt the devastating health impacts of chronic financial stress. Current literature openly acknowledges that there remains a significant gap in fully understanding the mechanisms through which chronic stress drives disease. As Lagraauw et al. [81] explicitly notes, while chronic stress is undeniably linked to acute coronary syndromes (ACS), existing human studies remain largely correlational, lacking sufficient exploration into the precise causal mechanisms driving disease. By leveraging the social-ecological model, this study provides a sound and comprehensive framework specifically tailored to expose and disrupt the systemic forces perpetuating financial stress within Black communities and their direct implications for cardiovascular health.

We begin our analysis of the social-ecological model at the policy level rather than the individual level. Research demonstrates that interventions at these higher social-ecological levels achieve broader public health impact with minimal individual burden [82]. Frieden [82] outlines a pyramid of interventions, emphasizing that strategies targeting socioeconomic determinants of health at the pyramid’s base yield the most substantial benefits. These policy-driven interventions significantly improve population health by altering contexts to facilitate healthier default decisions, thereby reducing reliance on individual effort [82].

## Policy level: screening & funding gaps

### Misclassification of chronic stress in CVD frameworks

A critical policy gap is the absence of routine screening for financial stress as a cardiovascular disease risk factor. Moran and colleagues, in the Jackson Heart Study involving 2,256 Black participants in the Jackson, Mississippi metropolitan area, underscored financial stress as a significant yet largely unrecognized risk factor for coronary heart disease among African Americans [6]. Despite these findings, cardiovascular medicine continues to marginalize psychological stressors within clinical guidelines [31]. Consequently, chronic financial strain is consistently overlooked during CVD risk assessment. Our latent class analysis further reveals this oversight: a subgroup of Black Catholics (Profile 1) exhibits both high financial stress and severely reduced Mental Component Summary scores (MCS)—clear biomarkers indicative of cardiovascular disease risk [8]. Yet, health systems frequently misclassify these indicators based solely on the chronic stress timescale, overlooking their established link to cardiovascular disease [8]. Such misclassification directly leads to inadequate risk identification and intervention. Eleazu [83] highlights this neglect, attributing it to a narrow research focus limited to single stress domains, thereby omitting comprehensive chronic stress assessments crucial for accurately evaluating cardiovascular risk. Research consistently warns of severe consequences resulting from overlooking chronic stress in in long-term cardiovascular frameworks. Sternthal et al. [84] argue that neglecting chronic stressors results in incomplete and inaccurate health risk evaluations. Similarly, the INTERHEART Study—among the most extensive global analyses of stress and myocardial infarction (MI)—reported that chronic stress increases MI risk by 2.1 times [85]. Further evidence explicitly ties chronic stress to accelerated atherosclerosis in African Americans, affirming its role in precipitating early cardiovascular damage [80,86].

Our structural equation modeling analysis confirms financial stress as a significantly superior predictor of chronic stress risk (CHRONRSK) compared to the Perceived Stress Scale (PSS), as evidenced by a stronger model fit (χ²(3)=3.978, p = 0.2639 versus the PSS model, χ²(3)=17.678, p = 0.0005). This indicates that generalized stress measurements such as PSS inadequately capture the socioeconomic stressors uniquely burdening Black communities [87]. Thus, accurately classifying Black populations experiencing elevated financial stress into appropriate high-risk categories is vital for meaningful intervention.

### Policy implications and call to action

Failure to identify chronic financial stress within current CVD models compromises risk stratification, delaying the low‑cost preventive therapies that could avert the first AMI and stroke events highlighted by Sandhu et al. (2022) [11]. This oversight denies at-risk Black subgroups, particularly Black Catholics, essential targeted interventions provided by the BaCE Pathway. Despite overwhelming evidence that Black Americans face the highest coronary heart disease (CHD) mortality rates among all ethnic groups [24], policy-level inertia persists.

To effectively address this disparity, we strongly recommend:

- Mandatory financial stress screening within standard CVD risk assessments to identify at-risk individuals early.
- Increased research funding focused explicitly on chronic financial stress and its role in exacerbating CVD among diverse Black subpopulations.
- Policy reforms that integrate psychosocial stress markers into clinical guidelines, in alignment with significant findings from studies like INTERHEART [31].
- Expanded research into social determinants, particularly financial stress, as critical drivers of cardiovascular disparities [6].

Sandhu et al. (2022) [11]. estimate that more than one million Americans experience a first acute myocardial infarction or ischemic stroke each year, in part because suboptimal risk identification limits timely, low‑cost preventive therapies. When chronic financial stress goes unrecognized—an omission that disproportionately affects Black communities—these diagnostic gaps can compound existing cardiovascular inequities.

## Discrimination as a community-level impact

### Racial bias intensifying financial burdens

Discrimination significantly magnifies chronic cardiovascular disease risk among Black Catholics. Our analysis reveals that Profile 1, representing individuals at greatest risk for CVD, experiences profoundly worse health outcomes, compounded by the highest reported levels of discrimination. Black Catholics reported notably higher discrimination scores (mean = 5.12) compared to Black Protestants (mean = 2.8) and Black Atheists/Agnostics (mean = 1.0). These stark disparities demand integrated tracking of financial stress and discrimination to ensure precise identification and targeted interventions.

Our latent class analysis explicitly supports identifying and tracking individuals within Profile 1, characterized by severe financial stress, poor mental health outcomes, and elevated discrimination levels (6.33 ± 2.40). Structural equation modeling (SEM) further confirms discrimination as a powerful predictor of chronic health risks among Black Catholics (β = 0.227, p < 0.001). Previous studies clearly link discrimination as a chronic stressor directly affecting physical health, notably through elevated blood pressure [88].

When the MCS node is excluded in a sensitivity test, financial strain and discrimination emerge as the dominant, independent driverThese results emphasize the urgent need for stress assessment frameworks sensitive enough to capture distinct disease expressions within Black subpopulations. Historically, medical research and public health initiatives have inaccurately treated the Black population as a monolithic group [21]. This oversimplification has repeatedly led to underdiagnosis and misclassification of discrimination and economic stress effects among Black Catholics. Adopting a targeted, subpopulation-focused analysis is crucial to accurately identify and address the unique intersections of financial stress and discrimination that contribute significantly to CVD risk.

### Challenging the Black–White “mental health paradox”

The frequently cited resilience of Black populations under stress, known as the Black–White Mental Health Paradox, collapses when examining specific subpopulations. Earlier researchers, including Williams [89], recommended subpopulation analyses as essential to accurately investigate this paradox. Our findings directly contradict the paradox narrative: Black Catholics demonstrated significantly higher stress and discrimination accompanied by lower Mental Component Summary (MCS) scores. White Catholics experiencing similar financial stress still reported on average higher MCS scores (41.98 vs. 40.81), highlighting the deep-seated disparities.

Existing literature indicates that life-course factors might explain contradictory mental health findings among Black adults [90]. However, our disaggregated subgroup analysis uncovers underlying systemic inequities clearly. Improved health outcomes for Black populations depend on effectively addressing and mitigating cumulative discrimination [21]. Tracking financial stress patterns within the social-ecological framework becomes vital for understanding how discrimination intensifies economic distress, driving adverse cardiovascular outcomes.

### Healthcare (institutional failures)

Analyzing financial stress through the social-ecological model allows us to scrutinize its interaction with healthcare experiences among Black Catholics. At the institutional level, financial stress significantly intensifies existing healthcare disparities, deepening systemic injustices faced by this community. Historical mistrust towards healthcare institutions among Black populations is rooted in well-documented unethical practices and racial biases [91]. Yet, building trust is critical for effective cardiovascular disease prevention and management.

A particularly compelling finding underscores provider racial concordance as crucial to patient satisfaction. Black patients consistently report superior healthcare experiences with Black healthcare providers [92]. This finding emphasizes an immediate need to increase Black healthcare professionals within facilities serving Black communities to mitigate biases and build essential trust. Our one-way ANCOVA analysis identified patient satisfaction as the only significant covariate influencing MCS (p < 0.001). Additionally, ANOVA results indicated that poor patient satisfaction explained 38.3% of variance in Mental Component Summary (MCS) scores, reinforcing the direct link between healthcare experiences and mental health outcomes. Studies consistently show discrimination in healthcare settings significantly deteriorates mental health, exacerbating both psychological and financial stress among Black patients [91].

Financial stress profoundly impacts healthcare trust and satisfaction. Black Catholics reported high dissatisfaction with healthcare when financial stress levels were elevated, suggesting economic strain significantly worsens healthcare experiences. This aligns with documented experiences of Black patients encountering racially biased treatment [93], deterring engagement and adherence to medical recommendations [24]. Recognizing how financial stress shapes patient satisfaction and trust is critical for effective healthcare interventions.

A central element of the BaCE pathway involves identifying financial stress patterns and tracking their effects across different domains. As individuals navigate this pathway, pinpointing high-risk areas becomes essential for successful intervention [30]. Establishing clear markers for early identification can significantly mitigate financial stress-related health risks.

Our PROCESS mediation analysis further supports this viewpoint, showing increased financial stress significantly elevates healthcare dissatisfaction, subsequently harming mental health (indirect effect = -0.3885, 95% CI [-0.4832, -0.3018]). This cyclical dynamic intensifies risks along the BaCE pathway: high financial stress erodes healthcare trust, worsening mental health, and accelerating cardiovascular disease progression. Addressing these institutional failures demands targeted action to eliminate racial biases, improve financial accessibility, and ensure equitable healthcare quality for Black patients.

### Individual level: physiological and cognitive consequences

Connecting financial stress and chronic stress explicitly to cardiovascular disease is crucial to revealing their devastating impact [14], previously hidden due to systemic misclassification. Our latent class analysis identified distinct subpopulation patterns, using targeted metrics to effectively pinpoint and support individuals at the highest risk. At the individual level, our data clearly show that Black Catholics experiencing the highest financial stress (mean = 5.79) also reported the lowest MCS scores (mean = 39.35) and lowest Physical Component Summary (PCS) scores (mean = 35.45). These findings stress the importance of examining disease progression beyond general racial categories. Broad approaches emphasizing acute events overlook essential variations, despite compelling evidence linking chronic stress to accelerated disease progression in specific subpopulations [86].

Reframing financial stress beyond simplistic views of budgeting or personal mismanagement is crucial at the individual level. Instead, the social-ecological model positions financial stress as a systemic issue influenced by broader socioeconomic forces. Stokols [94] criticized traditional health promotion programs for narrowly focusing on individual behaviors, ignoring critical environmental determinants. Traditional interventions, such as financial literacy programs, often fail because they emphasize individual responsibility and neglect upstream structural causes [95]. Cognitive flexibility training, conversely, equips individuals to recognize these systemic factors, promoting holistic and effective approaches to managing financial stress [96].

Our findings reveal a striking pattern in how participants perceive financial stress across the social-ecological model. Individuals experiencing high financial stress (Quartiles 3 & 4) overwhelmingly attributed their difficulties solely to personal, relational, or institutional factors, overlooking policy or community-level stressors. Conversely, those with lower financial stress (Quartiles 1 & 2) exhibited greater cognitive flexibility, recognizing systemic factors, such as policy and community issues, as contributing significantly to their financial hardships. Further, our analysis identified a clear negative correlation: as financial stress increased, individuals’ capacity to perceive broader systemic causes diminished, narrowing their focus to immediate interpersonal challenges. This narrowed perspective is deeply troubling, given that prolonged financial strain is a documented predictor of chronic health decline and increased mortality risk [30].

Understanding these patterns of financial stress perception is critical for reshaping intervention strategies. To effectively address financial stress, individuals must receive support to broaden their recognition of its systemic roots. This systemic perspective parallels the call by former American Psychological Association president de las Fuentes for “structural competence” in population mental‑health practice [20]. Specifically, she called for interventions addressing root causes deeply embedded in social structures. Our research underscores the potential effectiveness of cognitive flexibility training in enhancing awareness of financial stress as a multi-level societal issue rather than a purely personal problem. A study by Zhu [97] demonstrated the effectiveness of cognitive flexibility training in mitigating stressors. By enabling individuals to view financial strain through a social-ecological lens, we can develop sustainable interventions to mitigate financial stress and consequently reduce cardiovascular risk within marginalized communities. Social-ecological approaches have already proven effective in interventions aimed at improving cardiac health [23].

### Church and community networks: limited buffer

Typically, church networks serve as a critical buffer against financial stress within the Black Cardiovascular Ecological Pathway, offering substantial community support [51]. However, despite their capacity to provide immediate charitable assistance, our findings suggest financial stress is deeply systemic, extending far beyond personal economic predicaments. This systemic financial stress permeates multiple social-ecological levels, overwhelming the ability of traditional pastoral structures to provide effective long-term relief. Furthermore, reliance on charity can obscure governmental responsibility, allowing authorities to avoid adequately addressing systemic financial inequities and fulfilling their human rights obligations [98].

Our mediation analysis demonstrates that although being a Black Catholic initially appeared beneficial to mental health (Mental Component Summary score, MCS) when controlling for financial stress (b = 4.09, p = 0.0288, 95% CI [0.4260, 7.7527]), this positive impact was severely undermined by financial stress itself. The indirect effect of Black Catholic status on MCS through financial stress was negative and highly significant (b = -3.94, 95% CI [-5.5815, -2.4389]), clearly demonstrating how financial stress erodes the mental health benefits typically provided by religious affiliation. Financial stress accounted for 25.92% of the variance in MCS scores (R² = 0.2592, F(2,282) = 49.34, p < 0.001). Furthermore, financial stress exerted a significant negative influence on mental health (b = -0.53, p < 0.001), underscoring how financial stress severely diminishes the protective psychological and emotional support usually found within faith communities.

This finding is especially alarming given the traditional role faith-based institutions play as buffers against stress [51,99,100]. However, for Profile 1—the subgroup facing the highest stress levels—religious affiliation failed to offer significant protection against financial strain. The systemic distress experienced by this group surpasses the parameters of traditional charitable responses [98]. Given the systemic nature of financial stress, individual acts of charity fall profoundly short [98]. Instead, structural solutions aligned with cooperative economic models, historically championed by Catholic social teaching, are urgently required. These cooperative models prioritize shared ownership, communal investment, and human dignity above mere capital accumulation [101].

## Conclusion

We began this inquiry recognizing that Black Catholics exhibited distinct and concerning markers of cardiovascular disease risk, specifically elevated financial stress and notably low Mental Component Summary scores. Despite these clear risk indicators, standard cardiovascular frameworks failed to appropriately map these individuals toward necessary intervention pathways [8]. Instead, the chronic, persistent nature of their financial stress—contrary to “lifetime” stress timelines—diverted them onto pathways associated with heightened mortality risk. This misclassification occurs when interventions narrowly target the individual level within the social-ecological model, emphasizing behavioral adjustments while systematically neglecting the structural roots of financial strain.

Our structural equation modeling illuminated this issue further, showing that although low MCS scores were significant, their predictive power weakened substantially in the presence of discrimination, a fierce upstream determinant of health disparities. It is more convenient for healthcare and policy systems to confine Black financial distress within individual level parameters—addressed superficially with therapeutic interventions and medication—rather than confront the deeper institutional inequities fueling this distress. Such systemic misdirection is not merely an oversight—it represents a profound injustice.

We present this analysis in unequivocal terms to clarify how financial stress operates as a fundamental structural injustice driving chronic financial-stress risk profiles. Though discourse often gravitates toward individual explanations for economic hardship, our findings explicitly resist this limited perspective. By systematically identifying stress patterns and tracing them upstream, we redirect critical attention toward the structural determinants underpinning health disparities. The disproportionate cardiovascular burden carried by Black Catholics is not only a health crisis but a moral imperative. Allowing them to remain trapped within intervention models that ignore the systemic origins of financial stress is both ineffective and ethically untenable.

Addressing financial stress at its structural roots is essential for genuine progress in health equity. Only by confronting and dismantling these upstream injustices can we ensure meaningful and sustainable cardiovascular health interventions for marginalized communities. Accordingly, advancing cardiovascular equity will depend on developing comprehensive, socially just pathways that explicitly confront chronic financial stress and its structural determinants. Embedding such pathways within research, clinical practice, and policy is essential to reduce cardiovascular risk among Black Catholics and other marginalized populations.

## Limitations section

This study has several limitations. Firstly, the relatively small sample size may affect statistical power and limit the generalizability of findings to the broader Black Catholic community, particularly regarding financial stress, chronic stress-related risk, and mental health.

Secondly, the cross-sectional nature of the study prevents the establishment of causal relationships, as it captures data at one point in time. Longitudinal studies are necessary to better understand the temporal links between financial stress, mental health, and cardiovascular outcomes.

Additionally, although the focus on Black Catholics provides valuable subgroup insights, these results may not generalize to other Black populations or religious groups. Furthermore, the financial stress and mental health measurement scales used may not fully encapsulate the multifaceted complexities associated with intersectionality and socioeconomic influences.

This study also did not examine potentially influential factors such as regional variations, healthcare access disparities, and cultural perceptions regarding financial health. Such unmeasured confounders may significantly impact cardiovascular outcomes.

Despite these limitations, the current study provides a foundational basis for future research aimed at enhancing risk classification for Black Catholics, particularly concerning financial stress as a **potential driver** of cardiovascular disease. These findings underscore the critical need for improved risk categorization and more inclusive public health strategies.

## Future directions

### Advancing evidence-based financial stress interventions

Pope Francis, in Laudato Si’, aligns his vision of integral ecology with the principles of cooperative economics—a model prioritizing human well-being over capital interests [101]. Building upon our findings, the Black Catholic Health Research Institute has developed an evidence-based intervention designed to mitigate financial stress through immersive social-ecological simulations.

God’s Little World, a traveling experiential lab, simulates real-world financial stressors across various social-ecological levels. Participants collaboratively navigate both capitalist and cooperative economic scenarios, addressing financial challenges within family teams. Structured debriefings allow participants to reflect on their decision-making, enhance cognitive flexibility, and explore cooperative economic alternatives. Such educational simulations offer participants meaningful opportunities to directly engage with and proactively address societal issues [102].

To strengthen empirical assessment, our research incorporates heart rate variability (HRV) as a physiological indicator of financial stress [103]. HRV monitoring allows precise measurement of stress-related biological impacts, differentiating between capitalist and cooperative phases of the simulation. This approach will yield concrete, data-driven evidence on whether cooperative economic practices effectively alleviate financial stress for Black communities.

In 2026, God’s Little World will launch its pilot in the Diocese of Fort Worth, Texas, with plans for national expansion. This initiative systematically assesses participant stress levels, behavioral adaptations, and long-term interest in cooperative models. Combining quantitative data with participant insights, we aim to evaluate cooperatives’ practical viability as sustainable financial stress interventions. This innovative program represents a crucial step towards scalable, community-driven solutions, equipping individuals to address financial instability, advocate systemic change, and evaluate cooperative economics as a meaningful pathway forward.
